# Electrospray-Orbitrap Analysis of Nitrate and Sulfate Isotopologues in Natural Samples: Performance and Constraints

**DOI:** 10.1021/acs.analchem.6c00183

**Published:** 2026-08-31

**Authors:** Jack Saville, Julien Witwicky, Elsa Gautier, Alexis Lamothe, Nils Johannes Kuhlbusch, Becky Alexander, Andrew Schauer, Andrea Burke, Priyesh Prabhat, Eva Stüeken, Joshua Garber, Nicolas Caillon, Joël Savarino

**Affiliations:** † Univ. Grenoble Alpes, CNRS, IRD, Grenoble-INP, INRAE, IGE, Grenoble 38000, France; ‡ International Center for Isotope Effects Research, Nanjing University, Nanjing 210023, China; § University of Münster, Münster 48149, Germany; ∥ Thermo Fisher Scientific GmbH, Bremen 28199, Germany; ⊥ Department of Atmospheric and Climate Sciences, University of Washington, Seattle, Washington 351640, United States; # Department of Earth and Space Sciences, University of Washington, Seattle, Washington 351310, United States; ∇ School of Earth & Environmental Sciences, 7486 University of St Andrews, St Andrews KY16 9TS, United Kingdom

## Abstract

Traditional isotope ratio mass spectrometers are not optimally designed to analyze nonvolatile molecular solutes such as oxyanions or organics. Necessary sample conversion steps, often demanding micromole-scale samples, break chemical bonds and limit oxyanion analyses to elemental isotope ratios. Recent studies enthusiastically promote soft electrospray ionization coupled to high-resolution Orbitrap mass spectrometry (ESI-Orbitrap-MS) for intact-molecule isotopologue analysis. This combination uses widely accessible instrumentation to provide simultaneous multi-isotopologue and clumped isotope analyses using tens of nanomoles of dissolved sample. However, its isotopic capabilities have been evaluated primarily through technical assessments rather than on natural samples, despite the need for matrix sensitivity assessments and robust isotopic calibrations. Here, we establish a framework for natural-sample ESI-Orbitrap-MS isotopologue analysis of nitrate and sulfate, addressing matrix effects, isotopologue calibration, and instrumental stability. We demonstrate strong sensitivity to contaminants at concentrations 1000 times below target analytes and strong temperature-correlated drift over >30 min, highlighting the enhanced need for rigorous sample purification to suppress matrix effects, sample-reference bracketing to overcome drift, and isotopologue calibration to reduce biases and robustly quantify uncertainties. We achieve 0.4–1.3‰ precision and accurate measurements of δ^15^N-NO_3_^–^, δ^18^O-NO_3_^–^, Δ^17^O-NO_3_^–^, δ^34^S-SO_4_^2–^, and Δ^17^O–SO_4_^2–^ relative to traditional techniques, consuming only 20 nmol nitrate or 10 nmol sulfate. We resolve relative nitrate clumped isotope values in ice core samples (Δ^15^N^18^O-NO_3_^–^: ±2.3‰; Δ^18^O^18^O-NO_3_^–^: ±3.3‰) yet observe no correlations with bulk nitrate isotopes. These results establish practical guidelines for applying ESI-Orbitrap-MS to isotopologue and clumped isotope analysis of trace analytes in challenging sample matrices under real-world conditions.

## Graphical abstract

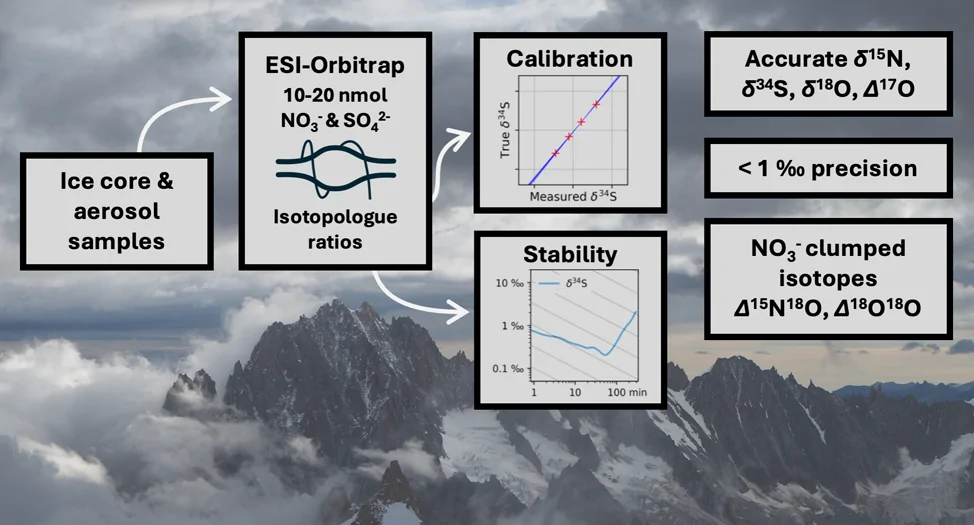

## Introduction

Precise, natural-abundance stable isotope ratio quantifications are useful tools across scientific disciplines. Over recent decades, analytical demands have expanded from bulk isotope ratios to more exotic analyses, including triple isotope systems, and compound-specific, clumped and position-specific isotope analyses across diverse sample types. The magnetic sector Isotope Ratio Mass Spectrometer (IRMS) remains the primary instrument for such work. However, its reliance on chemical conversions to gaseous species suitable for electron-impact ionization and its low mass-resolution restrict the application of IRMS to position-specific and clumped isotopes in nonvolatile molecular solutes such as oxyanions or organics. Moreover, large IRMS sample requirements for nonvolatile molecules (0.1–10 μmol) limit isotopic analyses of trace analytes and small samples such as paleoclimate archives or extraterrestrial samples.^1–3^ Specifically, N, S, and O isotope ratios in nitrate (NO_3_^–^) and sulfate (SO_4_^2–^) in aerosol, ice, water or soil samples, typically measured by a combination of denitrifier IRMS,^4^ Silver Salt Pyrolysis (SSP-) IRMS,^1^ Thermal Conversion/Elemental Analysis (TC/EA-) IRMS,^5^ and Multi-Collector Inductively-Coupled Plasma Mass Spectrometry (MC-ICP-MS)^6^ can diagnose the sources of N- and S-containing compounds in atmospheric or groundwater systems,^7–13^ and can elucidate critical zone biogeochemical processes,^14–22^ making them useful tracers in studies of water quality, palaeoclimate, nutrient cycles, archeology and forensics. However, the application of these methods to low-concentration and/or small-volume samples can be limited. For example, Δ^17^O–SO_4_^2–^ and Δ^36^S-SO_4_^2–^ IRMS measurements each require >1 μmol SO_4_^2–^, and are often impossible at the desired depth resolution in ice cores.^1,8^ Additionally, no single traditional technique can access both S and O isotope information in SO_4_^2–^, nor measure both δ^18^O–SO_4_^2–^ and Δ^17^O–SO_4_^2–^, and chemical conversion steps for IRMS are known to exchange or fractionate O isotopes in NO_3_^–^ and SO_4_^2–^, compromising measurement accuracy if poorly controlled.^23–25^ Besides, although elemental isotope ratios provide useful scientific insights, Walters et al. (2026) suggest that atmospheric NO_3_^–^ clumped isotopes–inaccessible to traditional techniques–encode additional useful atmospheric oxidation information.

The recently repurposed Electrospray-Orbitrap mass spectrometer (ESI-Orbitrap-MS) promises solutions to several limitations of traditional isotope methods for nonvolatile molecular solutes including NO_3_^–^ and SO_4_^2–^. By combining soft ionization with high mass resolution analysis,^27–29^ ESI-Orbitrap-MS promises high-sensitivity, compound-specific, simultaneous multi-isotopologue (isotopically substituted molecule) analysis of any readily ionizable molecule dissolved in an ESI-compatible solvent, eliminating chemical conversion steps, and enabling clumped isotope analysis of several nonvolatile analytes using nanomole sample amounts.^30–35^ ESI-Orbitrap-MS provides simple, almost-identical workflows for many dissolved analytes,^36^ while optional Collision-Induced Dissociation (CID) can enable analyte fragmentation–theoretically also allowing position-specific isotope analysis.^37,38^

Recent exploratory ESI-Orbitrap-MS oxyanion studies have accurately measured singly substituted isotopologues of nitrate, sulfate, phosphate, perchlorate, and methanesulfonate,^3,26,31,32,34,35,39–41^ and doubly substituted isotopologues of nitrate and sulfate,^31,34^ largely focusing on analysis of pure solutions. However, few studies have attempted ESI-Orbitrap-MS oxyanion isotopologue analysis of natural samples, which are challenged by poorly understood isotopic interferences with nonanalyte matrix components.^26,42^ While Walters et al. (2026) provided a matrix-matched natural sample preparation method for ESI-Orbitrap-MS nitrate isotopologue analysis and reproduced general IRMS δ^15^N-NO_3_^–^, δ^18^O-NO_3_^–^ and Δ^17^O-NO_3_^–^ isotopic patterns in aerosol samples, they reported significant mean biases and proportional biases for δ^15^N-NO_3_^–^ and δ^18^O-NO_3_^–^ between ESI-Orbitrap-MS and IRMS. Also, Walters et al. (2026) report nitrate clumped isotope variability of up to 10‰ between aerosol size bins, negative correlations with singly substituted isotopologues, and distinct clumping behaviors between rare isotope pairs. Such atmospheric nitrate clumped isotope variability, if replicable, could provide new insight into atmospheric oxidation pathways. However, since singly substituted isotopologues are involved in clumped isotope Δ-value calculations, poor correction of isotopologue scale distortions can lead to proportional biases and apparent correlations between clumped and singly substituted isotopologues. For example, an uncorrected δ^18^O^18^O-NO_3_^–^ scale contraction of 10% generates an apparent slope of −0.2 between δ^18^O-NO_3_^–^ and Δ^18^O^18^O-NO_3_^–^, even in the absence of true ^18^O–^18^O isotope clumping. The biases and negative correlations observed by Walters et al. (2026) could reasonably be the results of poor isotopologue calibrations, obscured by underestimated calibration-associated uncertainties. As with all IRMS techniques, the isotopic accuracy of ESI-Orbitrap-MS depends heavily on the isotope standards and the calibration protocol employed, yet reports and discussions of calibration-associated uncertainties are missing from the ESI-Orbitrap-MS literature. Unlike NO_3_^–^, ESI-Orbitrap-MS SO_4_^2–^ isotopologue ratios in natural samples have not yet been reported. SO_4_^2–^ isotopologues occupy a *m*/*z* range shared with several common anions, complicating their isotopologue analysis. Successful application of ESI-Orbitrap-MS to SO_4_^2–^ isotopologues in natural samples would validate the first method capable of simultaneously accessing S and O triple isotope information in SO_4_^2–^, requiring injection of approximately 10 nmol SO_4_^2–^. Compared to the current set of methods (MC-ICP-MS, SSP-IRMS, and TC/EA-IRMS, which require injections of approximately 5, 10^3^, and 10^3^ nmol for δ^34^S & Δ^33^S, Δ^17^O, and δ^18^O analyses, respectively), ESI-Orbitrap-MS would bring substantial improvements in both sensitivity and workflow simplicity, while supporting the broader application of ESI-Orbitrap-MS to isotopic analysis of more chemically diverse or challenging analytes. Furthermore, maintaining instrumental stability over the time scale of sample–reference alternation is crucial for precise isotopic measurements. However, the isotopic stability of ESI-Orbitrap-MS–an instrument not designed for high-precision analysis–remains unclear: several recent studies employed the relative standard error (RSE) metric and found no evidence of instrumental drift,^33–35,43^ while Hong et al. (2025) instead used the relative Allan deviation (RAD) metric, finding that ESI-Orbitrap-MS isotopologue ratios can drift on the time scale of a few minutes. These contrasting conclusions must be reconciled, to provide a realistic view of the isotopic precision achievable using ESI-Orbitrap-MS.

In this work, we investigate the performance of ESI-Orbitrap-MS for δ^15^N-NO_3_^–^, δ^18^O-NO_3_^–^, Δ^17^O-NO_3_^–^, δ^34^S-SO_4_^2–^, Δ^33^S-SO_4_^2–^ and Δ^17^O–SO_4_^2–^ measurements in natural samples. To this end, we adapt the semipreparative ion chromatography method^1,44^ to purify parallel NO_3_^–^ and SO_4_^2–^ extracts from ice core or aerosol samples for analysis by ESI-Orbitrap-MS. We report optimized ESI-Orbitrap-MS data acquisition, processing and multipoint isotopic calibration steps which generate isotopologue δ-values, triple isotope Δ-values and the relative clumped isotope values Δ^15^N^18^O-NO_3_^–^and Δ^18^O^18^O-NO_3_^–^. We then compare ice core NO_3_^–^ and aerosol SO_4_^2–^ isotopic measurements made using ESI-Orbitrap-MS and traditional methods, finding reduced NO_3_^–^ isotopic bias compared to Walters et al. (2026) and reporting the first natural sample SO_4_^2–^ isotopologue measurements. To portray the realistic capabilities of ESI-Orbitrap-MS under real-world conditions, we also discuss the impacts of observed matrix interferences, blanks, and memory effects, and characterize instrumental drift by introducing the relative Hadamard deviation (RHD) metric which is more useful than RSE or RAD for determining sample–reference comparison time scales. Finally, we propose guidelines for improving sensitivity, precision and accuracy and for isotopologue analysis of more challenging analytes or sample types, addressing a growing demand of the stable isotope community.

## Experimental Section

In this section, we describe a method for ESI-Orbitrap-MS NO_3_^–^ and SO_4_^2–^ isotopologue analysis of dilute aqueous solutions containing both analyte and nonanalyte ions, such as aerosol filter extracts, water, ice or snow samples. We apply the method to NO_3_^–^ from ice cores and SO_4_^2–^ from aerosol samples. The workflow, summarized in Figure 1, adapts the semipreparative ion chromatography sample purification method^1,44^ to produce methanolic NaNO_3_ and Na_2_SO_4_ solutions and combines the automated ESI-Orbitrap-MS isotopologue protocol of Kantnerová et al. (2024), with both *M*_0_ and *no-M*_0_ analyses as introduced by Neubauer et al. (2020) and Hilkert et al. (2021).

### Natural Samples

50 Mont-Blanc ice core samples were obtained from the 126 m core drilled at the Col du Dôme (45°50′ N, 6°51′ E, 4236 m a.s.l.) in 2016, as detailed by Lamothe et al. (2026). Compared to the urban aerosol samples previously analyzed for nitrate isotopologues by ESI-Orbitrap-MS, these ice core samples extend the range of accessible matrices to include dilute solutions containing higher proportions of long-lived atmospheric species. 12 Antarctic total aerosol samples were collected on glass-fiber filters using a high-volume aerosol sampler (GL-2000H, General Metal Works, Tisch Environmental) at Concordia station (75°06′ S, 123°20′ E, 3233 m a.s.l.), from January-December 2011, as detailed by Ishino et al. (2019).

### Natural Sample Preparation

Following a protocol similar to those described by Geng et al. (2013) and Walters et al. (2026), anions were separated, purified and converted to their sodium salt forms before solvent exchange by evaporation and redissolution in methanol. Samples and isotope standards were treated with suppressed-conductivity ion chromatography (IC) (Metrohm IC 850; Dionex AG15 preconcentration column; NaOH 10–50 mM eluent; Metrosep A Supp 16 separation column; H^+^-loaded suppressor (H_2_SO_4_ 100 mM; 95–98%, EMPROVE, Merck)), where NO_3_^–^ and SO_4_^2–^ peaks were isolated using a timed diverter valve. In this work, 100 nmol NO_3_^–^ or SO_4_^2–^ were targeted for separation. Unlike the offline equimolar-neutralization method of Walters et al. (2026), we passed the now-separated HNO_3_ and H_2_SO_4_ peaks directly after conductivity measurement through a cationic suppressor column (MSM, Metrohm) loaded with 150 mM NaCl (99.5%, Thermo Scientific Chemicals) and rinsed with ultrapure water. This accurately neutralized acidic anions and eliminated a manual operation, ensuring reproducibility and limiting possible contamination. Purified NaNO_3_ and Na_2_SO_4_ solutions were then collected using a fraction collector (XY-2, Seal) and IC measurements showed quantitative NO_3_^–^ and SO_4_^2–^ recovery (>97%). A solvent exchange was then performed to give methanolic solutions for analysis. For evaporation, 20 nmol NaNO_3_ or 10 nmol Na_2_SO_4_ solutions were transferred to 2 mL glass septum-vials (SureSTART level 3, Thermo Scientific). Evaporation in open vials was found to introduce interfering contaminants (see Matrix Effects section). Unlike previous studies who freeze-dried solutions, we heated vials to 60 °C while continuously flushing their headspace with pure N_2_ delivered via needles (Agani hypodermic, Terumo) at approximately 5 mL min^–1^ (evaporation rate approximately 0.5 mL h^–1^). Precipitated salts were redissolved at 45 μM in a mixture of methanol (CHROMASOLV HPLC-grade ≥ 99.9%, Honeywell) and formic acid (Optima LC/MS-grade, Fisher Chemical) (1000:1 v/v, henceforth referred to as *acidified MeOH*), and vortexed for 20 s. The target analyte concentration of 45 μM was selected to minimize memory effects and sample demand while limiting blank effects and isotopic concentration-dependence;^26,39^ 0.1% formic acid was added to buffer any pH differences and minimize isotopic concentration-dependence.^45^ Vials of running reference were similarly prepared by precipitating 45 nmol pure NaNO_3_ (USGS35, IAEA) or Na_2_SO_4_ (99%, Alfa Aesar, Thermo Scientific Chemicals) by evaporation, then reconstituting the salt in 1 mL acidified MeOH. Open-vial sample manipulation was performed under a laminar flow hood, and solutions were stored at 10 °C until analysis. Recovery of NO_3_^–^ and SO_4_^2–^ during evaporation, as determined by IC, was quantitative (>97%).

### Sample Introduction

For automated isotopologue analysis, an in-flow injection setup with a high-performance liquid chromatography (HPLC) pump and autosampler was used (Vanquish Neo, no separation column, Thermo Scientific), providing autonomous, reproducible multiday sequences following Kantnerová et al. (2024). As shown in Figure 1, each NO_3_^–^ (SO_4_^2–^) injection comprised 6 min of stable sample delivery, 4 min (9 min) to flush the system with eluent, and 2 min to automatically rinse the sample loop, for a total of 12 min (17 min) per injection. Typical injection sequences were structured as follows: 8 injections of running reference to condition the system, then alternating running reference injections and sample/isotope standard/blank injections, in randomized order, for a total of 6 bracketed injections for each sample and isotope standard vial.

**Figure 1:**
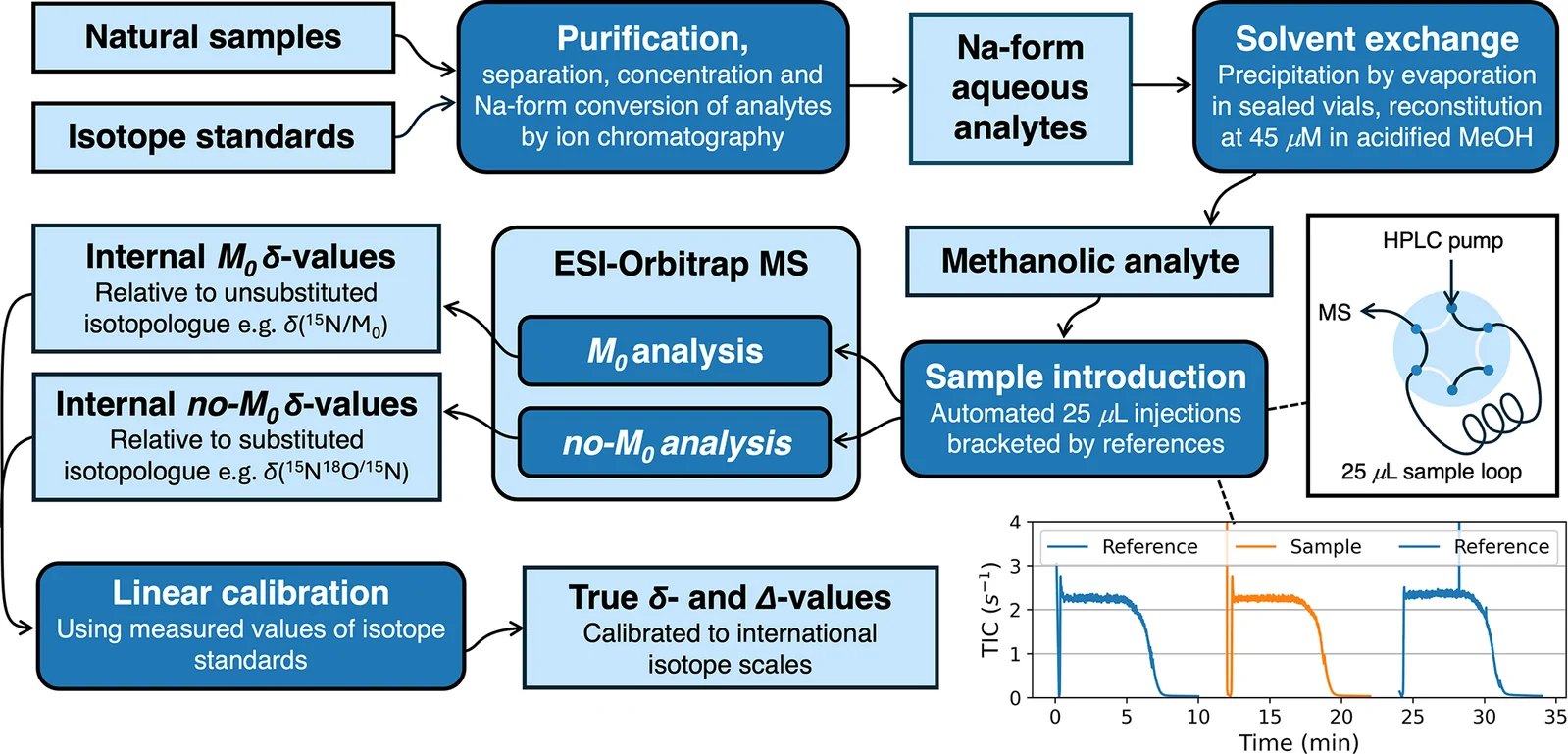
A flowchart summarizing the ESI-Orbitrap-MS acquisition workflow for isotopologue analysis of natural samples. Dark blue boxes represent the processes described in this section, while light blue boxes represent states. Sample–reference bracketing is achieved by automated in-flow injections using a six-way valve, with TIC signals depicted for NO_3_^–^ at bottom right. TIC = total ion current.

### Isotopologue Analysis

ESI-Orbitrap-MS (Orbitrap Exploris 240, OptaMax NG ion source, ESI-LOFLO needle insert, Thermo Scientific) was used for isotopologue analysis, using the instrumental parameters listed in Supporting Information Table S1. Method details can be found in Hilkert et al., (2021) and Kantnerová et al. (2024). ESI parameters were tuned before every experiment to optimize total ion current (TIC) stability (TIC relative standard deviation (RSD) target <10%, using one microscan for tuning), while keeping spray current <0.3 μA. Typical ranges for tuned ESI voltage and gas flows, and typical TIC and TIC RSD for analyte solutions are listed in Supporting Information Table S1. During multiday acquisition sequences, daily ESI stability checks were made, and sequences were paused for ESI retuning if TIC RSD was >10%. To improve the precision of minor NO_3_^–^ isotopologue ratios, *no-M*_0_ analysis sequences were made alongside *M*_0_ sequences, following Hilkert et al. (2021). The masses and abundances of accessible NO_3_^–^ and HSO_4_^–^ isotopologues are tabulated in Supporting Information Table S2. For SO_4_^2–^, only *M*_0_ analyses are reported in this study, due to problematic matrix effects in *no-M*_0_ analyses of natural samples (see Results and Discussion section).

### Blanks

Blanks were prepared from ultrapure water and acidified MeOH at several stages of sample preparation. *Procedural blanks* prepared alongside samples from the IC step onward showed conductivity signals during separation representing (0.04 ± 0.02) nmol NO_3_^–^ and (0.3 ± 0.3) nmol SO_4_^2–^, (±1 s.d.)–negligible relative to typical IC loads of 100 nmol; *evaporation blanks* were prepared alongside samples from the solvent exchange step onward; *solvent blanks* were vials containing acidified MeOH; and the *eluent blank* refers to the baseline TIC measured by ESI-Orbitrap-MS during pure eluent infusion. Measured TIC values for each type of blank, summarized in Table 1, allowed qualitative assessment of contamination levels and sources in our laboratory. Note while TIC generally increases with increasing analyte concentration, TIC saturates at high analyte concentrations and is matrix-sensitive, meaning TIC may overestimate blank.^46^ Samples display TIC signals 20–150 times greater than procedural blanks, meaning the isotopic blank effect is inferior to analytical precision. Nevertheless, this exercise allows us to qualitatively identify small NO_3_^–^ contributions from the vials, the IC and the solvent exchange step, and a small SO_4_^2–^ contribution from the vials and the solvent exchange step. Blank levels were too low for quantification of their isotopic contributions: blanks were therefore used for quality control purposes, rather than for quantitative corrections. For isotopologue applications of ESI-Orbitrap-MS in valuable samples, smaller sample requirements may be desirable. Robust analysis of <10 nmol samples may require reduction of procedural blank levels, by sample preparation in a dust-free environment, automation of sample preparation steps, or simplification of the IC setup.

**Table 1:** Summary of Typical Total Ion Currents (TIC) Observed in NO_3_^–^ and HSO_4_^–^ *M*_0_ Analyses, for Blanks and Samples

	eluent blank	solvent blank	evaporation blank	procedural blank	typical sample
**Description**	HPLC eluent, after hours of uninterrupted infusion	Acidified MeOH	1 mL ultrapure water, evaporated and redissolved in 450 μL acidified MeOH	40 mL ultrapure water, prepared following full procedure	Ice core (NO_3_^–^) or aerosol (SO_4_^2–^) sample, prepared following full procedure
**NO**_**3**_^**–**^ ***M***_**0**_ **TIC (× 10** ^**6**^ **s**–^**1**^ **)**	0.1	1–2	2–4	2–5	100–300
**HSO**_**4**_– ***M***_**0**_ **TIC (× 10** ^**6**^ **s** ^**–1**^ **)**	0.1	1–2	8–9	8–9	300–500

### Memory Effects

One disadvantage of analyzing intact oxyanions versus neutral gases is the increased likelihood for memory effects via adsorption of solutes onto capillary walls. At 45 μM, SO_4_^2–^ solutions exhibited greater carryover between injections than NO_3_^–^ solutions, indicating stronger adsorption to capillary surfaces. To minimize the consequent memory effects, the flushing time between SO_4_^2–^ injections was increased from 4 to 9 min. Memory effects were quantified by inspection of isotopologue ratio time-variability in stacked acquisitions, a similar approach to Wang et al. (2024): NO_3_^–^ displayed no detectable memory effect, while, for SO_4_^2–^, stacked acquisitions implied that (6 ± 2)% of the isotopic signal in injection *n* originated from injection *n*-1 (see methodology in Supporting Information Text S1 and Figures S1–S2). Given samples had approximately equal SO_4_^2–^ concentrations, memory effects were constant between samples, inducing scale contractions in bracketed SO_4_^2–^ δ-values. In addition, because measurement of the bracketing reference preceding sample injection *n* was sensitive to the isotopic composition of injection *n*-2, bracketed SO_4_^2–^ δ-values depended weakly on the identity of neighboring samples. Each vial was analyzed multiple times in random order, so this manifests as δ-value precision losses of ϕ × 0.25 × $\sqrt{2}$ × Σ ≈ 0.02Σ, where Σ is the δ-value standard deviation of all samples and isotope standards, and ϕ is defined in Supporting Information Text S1. In these SO_4_^2–^ analyses, this amounted to <0.15‰, and no correction attempt was made.

### Data Processing

Isotopologue peak intensities and metadata were extracted from *.raw* files using IsoX software (Version 2022, Thermo Scientific). Subsequent data treatment was performed using in-house notebooks, described below, summarized in Figure 2, and archived on Zenodo (DOI: http://dx.doi.org/10.5281/zenodo.19495112).

(1) For each autosampler injection, a time filter (0.5–6 min) and then a TIC threshold filter (scans with TIC outside median TIC ± 20% excluded) were used to trim the injection plateau and remove short periods of ESI instability.
(2) Isotopologues detected in below 99% of scans were ignored. This avoided overestimation of small isotopologue ratios (such as ^36^S/*M*_0_), whose peaks approach the noise band and can be undetected in some scans.
(3) Scans representing extreme high or low numbers of ions (TIC × IT) can give inaccurate isotopologue ratios.^47,48^ To remove outlying scans from δ-value calculations, several TIC × IT filtering criteria were tested. Sigma-clipping at 2σ or 3σ, and filtering using mean ±5%, or mean ±10% thresholds, all produced bracketed NO_3_^–^ and SO_4_^2–^ δ-values which agree within 0.1‰. To filter outliers while discarding <0.01% of scans, a mean TIC × IT ± 10% threshold filter was applied.
(4) Average isotopologue ratios for each autosampler injection were computed using the “*ions.incremental*” parameter (an estimation of the number of each isotopologue ion in the Orbitrap, calculated by IsoX as described by Kantnerová et al. (2024)). Arithmetic mean scan ratio, geometric mean scan ratio and the ratio of summed ions over all scans were found to give bracketed δ-values consistent to within 0.03‰. For simplicity, arithmetic mean was used.
(5) Internal δ-values were calculated for every bracketed sample or isotope standard, for each available isotopologue ratio, using eq 1

1 $$\delta Y_{i}=\frac{2R_{sample}^{Y_{i}}}{R_{ref-}^{Y_{i}}+R_{ref+}^{Y_{i}}}-1$$

where *R* ^Y_*i*_^ is the Y*_i_*/Y_0_ isotopologue ratio, Y_0_ is the unsubstituted isotopologue in *M*_0_ acquisitions or the ^15^N isotopologue in NO_3_^–^ *no-M*_0_ acquisitions, and ref– and ref+ refer to the two neighboring running reference injections. As indicated in Figure 2, the mean and standard error, σ_SEM_ = σ/ $\sqrt{N}$ , of the *N* = 6 replicate injections for each sample or isotope standard gave internal δ-values and associated precisions.

**Figure 2:**
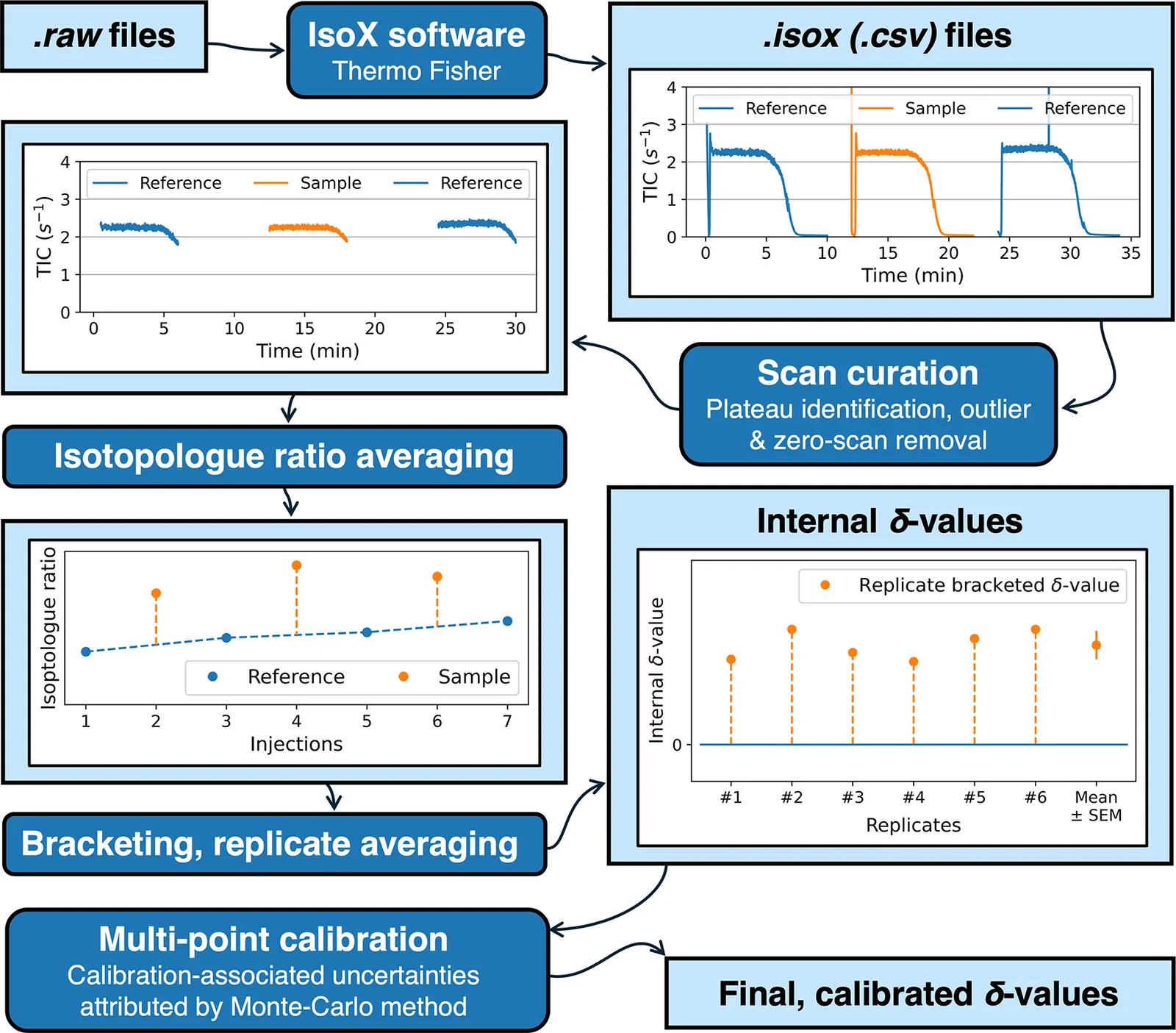
A flowchart summarizing the treatment steps used to transform raw ESI-Orbitrap-MS data to calibrated isotopologue δ-values. Dark blue boxes represent computational processes performed using IsoX software and custom notebooks, while light blue boxes represent states. TIC = total ion current; SEM = standard error of the mean δ-value.

### Isotope Standards and Calibration

Accurate stable isotope measurements require calibration to correct any systematic isotope fractionation during sample preparation and analysis. While sample-reference bracketing combats instrumental drift, isotope standards which have been treated identically to samples are also needed to correct matrix-dependent effects. Adequate correction of isotope scale distortions requires at least two isotope standards per isotopologue ratio, and comparison of isotopic measurements between laboratories requires the isotope standards to have established values on international isotope scales. The isotope standards used in this work, summarized in Table 2, were used to calibrate internal measurements to give δ-values on isotopologue scales which share the bulk O, N, and/or S isotope compositions of VSMOW, AIR and/or VCDT scales and assume stochastic intramolecular distributions. All standards were obtained in solid form and dissolved in ultrapure water to give 100 mM stock solutions, which were stored at 4 °C. For ESI-Orbitrap-MS, soluble isotope standards are required. While existing soluble standards are well characterized for δ^15^N-NO_3_^–^, δ^18^O-NO_3_^–^, Δ^17^O-NO_3_^–^, δ^34^S-SO_4_^2–^, Δ^33^S-SO_4_^2–^ and Δ^17^O–SO_4_^2–^, few soluble standards with published δ^18^O–SO_4_^2–^ values exist. To provide a full complement of δ-values, we obtained a new series of Na_2_SO_4_ standards (SodSul-2, SodSul-4, SodSul-6, SodSul-8) and performed an interlaboratory comparison to characterize their δ^34^S-SO_4_^2–^, Δ^33^S-SO_4_^2–^ and δ^18^O–SO_4_^2–^ by EA-IRMS, MC-ICP-MS, and SSP-IRMS. Table 2 lists provisional δ-values and Δ-values of these new standards, while their provenance, measured values, and analytical details are provided in Supporting Information Text S2. These new standards are available from the University of Washington IsoLab (isolab.ess.washington.edu). Clumped isotope standards for oxyanions do not yet exist. To bypass the need for absolute characterization of standards, we present relative clumped isotope values by assuming intramolecular stochasticity in USGS32, USGS34 and USGS35. This necessarily false assumption allows a provisional multipoint calibration and corrects scale distortions for double-substituted isotopologues, which could otherwise lead to bulk-isotope signals appearing in clumped isotope Δ-values. Future reprocessing of our data with absolute clumped isotope measurements of isotope standards would attribute absolute Δ-values to our samples.

**Table 2:** Accepted Values for NO_3_^–^ and SO_4_^2–^ Isotope Standards Used in This Study^a^

	NO_3_^–^reference values (‰)		
quantity (scale)	USGS32	USGS34	USGS35
**δ** ^**15**^ **N-NO**_**3**_^**–**^ **(AIR)**	180.0 ± 0.0	–1.8 ± 0.1	2.7 ± 0.1
**δ** ^**18**^ **O-NO**_**3**_^**–**^ **(VSMOW)**	25.7 ± 0.2	–27.9 ± 0.3	57.5 ± 0.3
**Δ** ^**17**^ **O-NO**_**3**_^**–**^ **(VSMOW)**		–0.3 ± 0.2	21.6 ± 0.2
**Δ** ^**15**^ **N** ^**18**^ **O-NO**_**3**_^**–**^	0.0 ± 0.0^†^	0.0 ± 0.0^†^	0.0 ± 0.0^†^
**Δ** ^**18**^ **O** ^**18**^ **O-NO**_**3**_^**–**^	0.0 ± 0.0^†^	0.0 ± 0.0^†^	0.0 ± 0.0^†^

	SO_4_^2–^reference values (‰)							
	SMIF-1	SMIF-2	Sulf-α	Sulf-ε	SodSul-2	SodSul-4	SodSul-6	SodSul-8
**δ** ^**34**^ **S-SO**_**4**_^**2–**^ **(VCDT)**	10.3 ± 0.2	21.5 ± 0.3			11.2 ± 0.5	0.9 ± 0.2	11.0 ± 0.1	–9.8 ± 0.1
**Δ** ^**33**^ **S-SO**_**4**_^**2–**^ **(VCDT)**	9.5 ± 0.1	11.4 ± 0.1	0.0 ± 0.1^†^	0.0 ± 0.1^†^	0.1 ± 0.1	0.0 ± 0.1	0.0 ± 0.1	0.0 ± 0.1
**δ** ^**18**^ **O–SO**_**4**_^**2–**^ **(VSMOW)**			–7.2 ± 0.6	–6.7 ± 1.2	7.1 ± 0.4	7.6 ± 0.3	14.8 ± 0.3	12.0 ± 0.5
**Δ** ^**17**^ **O–SO**_**4**_^**2–**^ **(VSMOW)**			0.9 ± 0.2	7.0 ± 0.2				

a USGS32, USGS34, USGS35, SMIF-1 and SMIF-2 values were determined from interlaboratory comparisons.^1,49^ Sulf-α and Sulf-ε oxygen isotope values were determined by gold-cup SSP-IRMS;^12,23^ values for the new standards SodSul-2, SodSul-4, SodSul-6 and SodSul-8 are the provisional results of our interlaboratory comparison detailed in Supporting Information Text S2; values marked † assume stochastic or mass-dependent isotope compositions.

In NO_3_^–^ *M*_0_ and *no-M*_0_ analyses, USGS32, USGS34 and USGS35 were used to calibrate δ­(^15^N/M_0_), δ­(^17^O/M_0_), δ­(^18^O/M_0_), δ­(^17^O/^15^N), δ­(^18^O/^15^N), δ­(^15^N^18^O/^15^N) and δ­(^18^O^18^O/^15^N), while, in SO_4_^2–^ *M*_0_ analyses, SMIF-1, SMIF-2, Sulf-α, Sulf-ε, SodSul-4 and SodSul-8 were used to calibrate δ­(^33^S/M_0_), δ­(^34^S/M_0_), δ­(^17^O/M_0_) and δ­(^18^O/M_0_). Henceforth, isotopic substitution notation refers to isotopologues (e.g., ^15^N^18^O = ^15^N^18^O^16^O_2_^–^). In each acquisition sequence, three vials (18 injections) of each standard were prepared and analyzed alongside samples. For each isotopologue ratio, the 18 measurements of each standard were averaged, and linear regressions were performed between measured and reference δ-values. Calibrated sample δ-values and calibration-associated uncertainties were assigned using a Monte Carlo scheme, using 1000 simulations each perturbing δ-values using normal distributions characterized by their standard errors to automatically assign greater calibration-associated uncertainty to poorly constrained regions of the calibration range. Additional details on the calibration protocol are provided in Supporting Information Text S3. After calibration, the mean and standard error, σ_SEM_, of the six replicate values for each sample gave the δ-value and its precision. The calibration-associated uncertainties, σ_MC_, reflected a systematic offset applicable to all samples in the sequence. The overall uncertainty on each sample was given by σ_tot_^2^ = σ_SEM_^2^ + σ_MC_^2^. Additionally, calibrated NO_3_^–^ δ-values from *M*_0_ and *no-M*_0_ analyses were combined to give δ-values for ^15^N^18^O and ^18^O^18^O isotopologues and more precise δ-values for ^15^N, ^17^O and ^18^O isotopologues, all with respect to the *M*_0_ isotopologue. These calculations are presented in Supporting Information Text S4. Finally, triple isotope and clumped isotope Δ-values were calculated using the approximations in Table 3, where clumped isotope formulas are derived from Eiler (2007).^50^

**Table 3:** Triple Isotope and Clumped Isotope Δ-Value Approximations Used in This Work^a^

value	formula
Δ^17^O	$\delta^{17}O-0.52\times \delta^{18}O$
Δ^33^S	$\delta^{33}S-\left[{\left(\delta^{34}S+1\right)}^{0.515}-1\right]$
Δ^15^N^18^O	$\frac{\delta^{15}N^{18}O+1}{(\delta^{15}N+1)\left(\delta^{18}O+1\right)}-1$
Δ^18^O^18^O	$\frac{\delta^{18}O^{18}O+1}{{\left(\delta^{18}O+1\right)}^{2}}-1$

a Note the distinction between triple isotope and clumped isotope Δ-values.

Typical calibration curves are presented in Figure 3: for isotopologue ratios with 3 or more calibration standards, strong linearity (*R* ^2^ > 0.997) was observed between measured internal δ-values and reference values, with typical residuals <1‰ for isotope standards and typical mean calibration slopes Δ­(reference value)/Δ­(internal value) in the ranges 0.98–1.10 for NO_3_^–^ *M*_0_, 1.05–1.12 for NO_3_^–^ *no-M*_0_, and 0.92–1.36 for HSO_4_^–^ *M*_0_. Calibration slopes were observed to vary mass-independently between isotopologues, necessitating independent calibrations of ESI-Orbitrap-MS δ-values before clumped or triple isotope Δ-value calculations. No nonlinear ESI-Orbitrap-MS behavior was observed across the studied isotopic ranges. The calibration-associated uncertainties attributed to the samples in this study were <0.2‰ for δ­(^15^N/*M*_0_), δ­(^33^S/*M*_0_) and δ­(^34^S/*M*_0_); 0.3–0.5‰ for δ­(N^18^OO_2_^–^/*M*_0_), δ­(^17^O/^15^N) and δ­(^18^O/^15^N), where samples were at times 13‰ outside the isotopic range of the standards; and 0.7–1.4‰ for δ­(N^17^OO_2_^–^/*M*_0_), δ­(^15^N^18^O/^15^N), δ­(^18^O^18^O/^15^N), δ­(^18^O-HS^18^OO_3_^–^/*M*_0_) and δ­(HS^17^OO_3_^–^/*M*_0_), where samples were at times 25‰ outside the isotopologue ratio range of the standards and where isotope standard ESI-Orbitrap-MS δ-values and/or reference δ-values carried substantial uncertainties. This robust, multipoint linear calibration protocol extends the 1-point and 2-point calibrations applied by Hilkert et al. (2021) and Walters et al. (2026) while additionally quantifying calibration-associated uncertainties. Such uncertainties are minimized by selecting precisely characterized isotope standards covering a suitably wide range of isotopic compositions, and by making precise ESI-Orbitrap-MS isotopologue ratio measurements.

**Figure 3:**
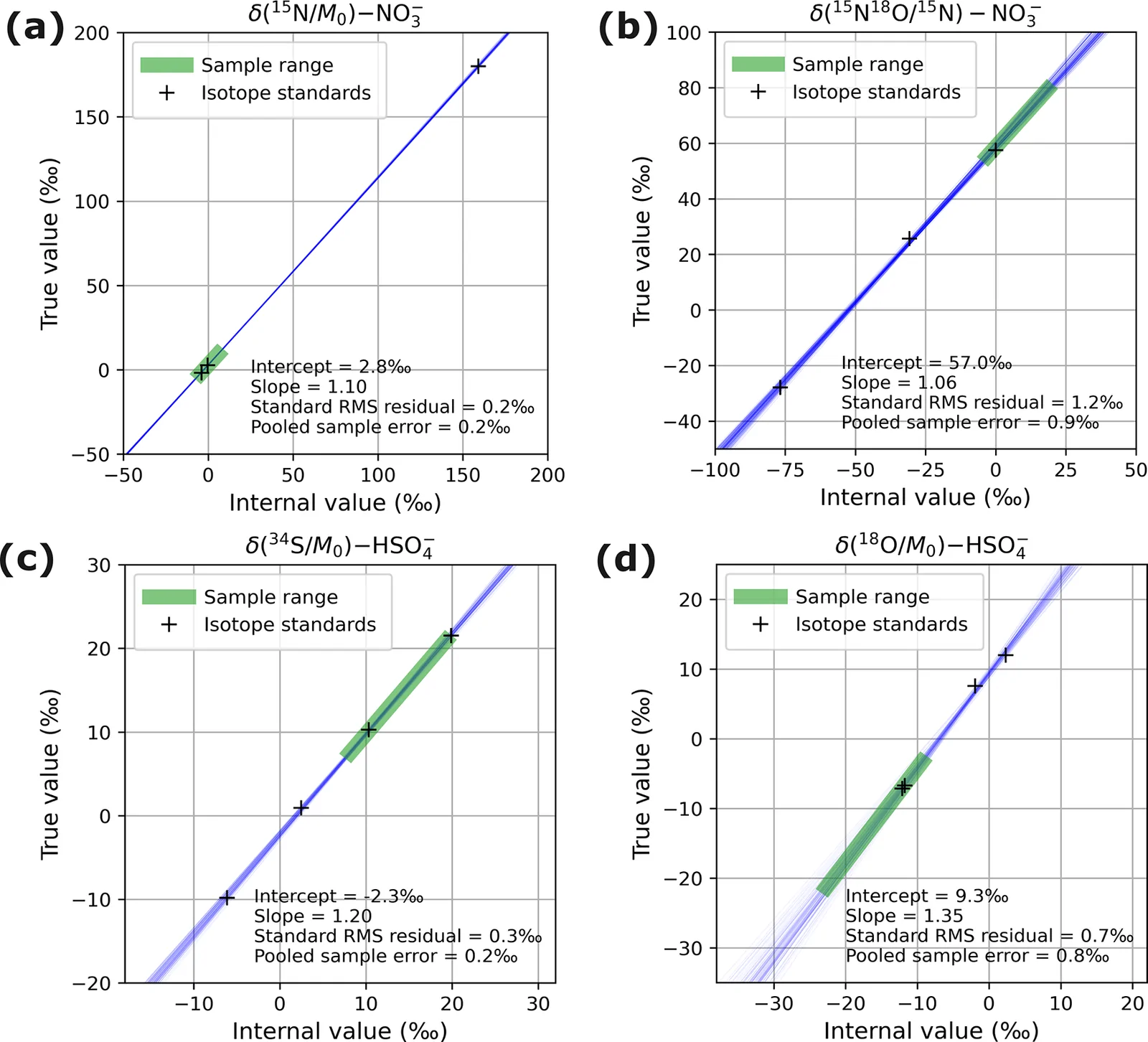
Examples of isotopologue calibrations for (a) NO_3_^– 15^N/*M*_0_, (b) NO_3_^– 15^N^18^O/^15^N (*no-M*_0_), (c) HSO_4_^–^^34^S/*M*_0_, and (d) HSO_4_^–^^18^O/*M*_0_. The blue lines represent linear regressions in the Monte Carlo scheme; their spread indicates calibration-associated uncertainty as a function of δ-value. Labeled intercepts and slopes are mean values; Standard RMS residual = root-mean-square residual for isotope standards; Pooled sample error = average calibration-associated uncertainty transferred to the samples in this study. Similar plots for other isotopologue ratios are provided in Supporting Information Figures S3–S5.

### Traditional Oxyanion Elemental Isotope Methods

The same samples were previously analyzed using denitrifier IRMS for δ^15^N-NO_3_^–^, δ^18^O-NO_3_^–^, and Δ^17^O-NO_3_^–^,^4^ SSP-IRMS for Δ^17^O–SO_4_^2–^,^1^ and MC-ICP-MS for δ^34^S-SO_4_^2–^ and Δ^33^S-SO_4_^2–^.^52^ Details of these previous analyses are presented by Lamothe et al. (2026) and Ishino et al. (2021, 2019, 2017).

## Results and Discussion

In this section, we first discuss observed impacts of matrix effects and instrumental stability on NO_3_^–^ and SO_4_^2–^ isotopologue ratio precision and accuracy. We then present a comprehensive intercomparison between NO_3_^–^ and SO_4_^2–^ isotope measurements obtained with ESI-Orbitrap-MS, IRMS and MC-ICP-MS on the same ice core and aerosol samples and discuss NO_3_^–^ clumped isotope measurements made on ice core samples.

### Matrix Effects

While the effect of contamination by target compounds was easily determined (see Experimental Section), the effects of nontarget compounds in ESI-Orbitrap-MS sample solutions (matrix effects) were more difficult to ascertain. Eiler et al. (2017) suggested three styles of matrix effect on Orbitrap isotopologue measurements: type 1 and type 2 “bulk” interferences altering the isotopic fractionation occurring in the ion source and quadrupole, respectively, and type 3 interferences due to coaccumulation of ions in the Orbitrap analyzer. Recent studies have attributed ion suppression–but not isotopic biases–to type 1 and 2 “bulk” matrix interferences^42^ and have reported only minor (0–3‰) isotopologue biases under high concentrations of salts or organics.^26^ Type 1 and 2 contaminants can have *m*/*z* outside the analysis mass window, hindering their identification postacquisition; however, sample purification by IC suppressed their isotopic impacts. In contrast, type 3 interferences–visible in Orbitrap mass spectra–have been reported to substantially degrade isotopic accuracy if baseline-resolved nontarget ions compose >10% of TIC^42,54^

During method development, two different type 3 interferences were observed, which arose from native coeluting compounds and contaminants introduced during sample preparation and weren′t observed for pure analytes directly prepared in methanol. First, as shown in Figure 4a,b, attempts to perform HSO_4_^–^ *no-M*_0_ analyses on aerosol samples were hindered by the interference of two nonanalyte spectral peaks in the same *m*/*z* range as substituted sulfate isotopologues (*m*/*z* = 98.9491 and 100.9458, attributed to ^35^ClO_4_^–^ and ^37^ClO_4_^–^), which accounted for 0–9% and 0–3% of *no-M*_0_ TIC in Antarctic aerosol samples, were baseline-resolved from all HSO_4_^–^ isotopologue peaks, and were not present in pure SO_4_^2–^ materials. Repeat preparations of the same samples found the same pattern of contamination, possibly caused by elution of ClO_4_^–^ (or a precursor subsequently converted to ClO_4_^–^) alongside the SO_4_^2–^ fraction. The δ-values for isotopologue ratios involving ^34^S- and ^18^O-substituted isotopologues differed by 0–30‰ between *M*_0_ and *no-M*_0_ acquisitions, while Δ^36^S was measured 0–40‰ lower than expected (see Supporting Information Figure S6). Strong proportionality was observed between *no-M*_0_ ClO_4_^–^ spectral peak intensities and M + 2 and M + 4 isotopologue biases, while no biases were apparent for M + 1 isotopologue ratios, implying that only isotopologues with the same integer *m*/*z* value as the contaminant were affected by this interference. Second, as demonstrated in Figure 4c,d, tests of solvent exchange by evaporation in open vials at 60 °C during method development were found to introduce an unidentified compound into some NO_3_^–^ samples which appeared as a peak emerging from the spectral noise in a small fraction (ca. 5%) of Orbitrap scans (*m*/*z* = 62.9891; signal/noise when peak detected = 2.2 ± 0.2). This contaminant peak was not baseline-resolved from ^15^NO_3_^–^ and N^17^OO_2_^–^ isotopologue peaks, and its detection coincided with 10–20‰ negative biases in measured δ^15^N-NO_3_^–^ and Δ^17^O-NO_3_^–^.

**Figure 4:**
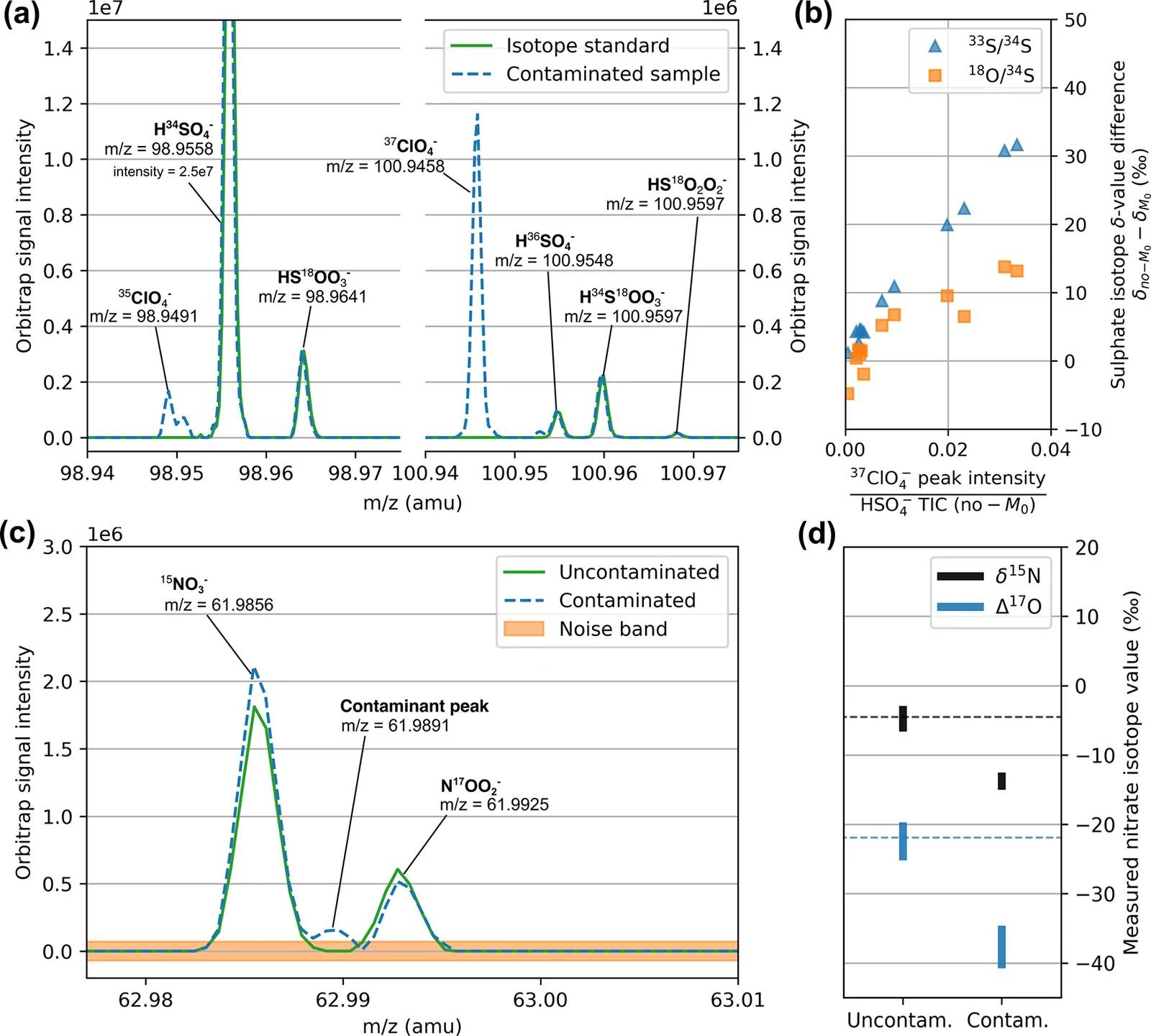
Representative ESI-Orbitrap-MS mass spectra demonstrating the contaminants suspected to have caused the type 3 matrix interferences. (a) A comparison between *no-M*_0_ spectra for an uncontaminated sulfate isotope standard and a representative contaminated Antarctic aerosol sample, where large spectral peaks attributed to ^35^ClO_4_^–^ and ^37^ClO_4_^–^ were observed. Note the different *y*-axis scales for the two spectral regions. (b) The relationship between the fraction of HSO_4_^–^ *no-M*_0_ TIC taken up by ^37^ClO_4_^–^ and the observed isotopologue ratio difference between *M*_0_ and *no-M*_0_ acquisitions. (c) A comparison between *M*_0_ spectra for uncontaminated and contaminated vials of a nitrate isotope standard, both prepared by evaporation in open vials, where a small contaminant peak is present between ^15^NO_3_^–^ and N^17^OO_2_^–^ isotopologue peaks in a small fraction of scans. No obvious chemical identity was attributed to this peak. (d) The corresponding ranges of δ^15^N and Δ^17^O values measured over 6 injections of an uncontaminated and a contaminated vial of a nitrate isotope standard.

To synthesize, we observed degraded ESI-Orbitrap-MS isotopic accuracy in the presence of coaccumulating contaminants with the same integer mass as analyte isotopologues, suggesting that >10‰ isotopic biases can be induced both when nonbaseline-resolved contaminant peaks are a similar amplitude to spectral noise, and when baseline-resolved contaminant peaks represent >1% of TIC – a lower threshold than that suggested by Hofmann et al. (2020) and Machado et al. (2026). Baseline-resolved interferences caused isotopic biases in *no-M*_0_, but not in *M*_0_ analyses, likely due to the amplified space-charge of the contaminant peak affecting the size of proximal isotopologue peaks^47^–an effect not reduced by increasing Orbitrap mass resolution. In each case, isotopic biases were induced by specific contaminants with spectral peaks over 3 orders of magnitude smaller than NO_3_^–^ or HSO_4_^–^ *M*_0_ peaks. The impacts of low-concentration contaminants in specific *m*/*z* ranges hinders generalized matrix effect investigations: likely interferences will depend strongly on the target analyte, sample matrix and laboratory environment. While preparing contaminant-free samples is the most robust strategy to avoid matrix effects, this is rarely achievable for natural samples. Instead, residual coeluting and coaccumulating species can be identified by inspection of separation chromatograms and ESI-Orbitrap-MS spectra, by monitoring spray current and TIC stability, and by flagging incoherences between *M*_0_ and *no-M*_0_ data. The impacts of identified contaminants on target isotopologue ratios should be quantitatively assessed, with dust-free sample preparation environments or more selective extraction techniques such as multistage IC applied as necessary.

### Instrumental Stability

ESI-Orbitrap-MS combines an instability-prone ionization process with a mass spectrometer not designed for precise abundance ratio measurements. Nevertheless, ESI-Orbitrap-MS has been successfully applied to isotopologue ratio measurements for several molecules, typically by maximizing Orbitrap analyzer ion throughput to improve statistical power. However, isotopic precision only follows shot-noise limits if sample-reference bracketing is performed on time scales short enough to compensate for any instrumental drift. Therefore, bracketing time scale selections must be guided by instrumental drift observations. Several studies used relative standard error (RSE) of continuous ESI-Orbitrap-MS data to imply that instrumental drift does not impact isotopic precision.^33–35,43^ However, Hong et al. (2025) recently used relative Allan deviation (RAD) to identify ESI-Orbitrap-MS methanesulfonate isotopic drift with time scales of a few minutes. These conflicting conclusions obscure the realistic prospects of ESI-Orbitrap-MS for precise isotopic analysis. To reconcile the apparent contradictions and select appropriate bracketing time scales, we conducted similar tests on 12 h ESI-Orbitrap-MS NO_3_^–^ and HSO_4_^–^ acquisitions. As summarized in Table 4, RHD, unlike RAD, is insensitive to linear drift, so is well-suited for determining reference–sample–reference bracketing time scales. RSE, RAD, and relative Hadamard deviation (RHD) were calculated for each isotopologue ratio following the protocol described in Supporting Information Text S5; the results are presented in Figures 5a–d and S7–S8.

**Figure 5:**
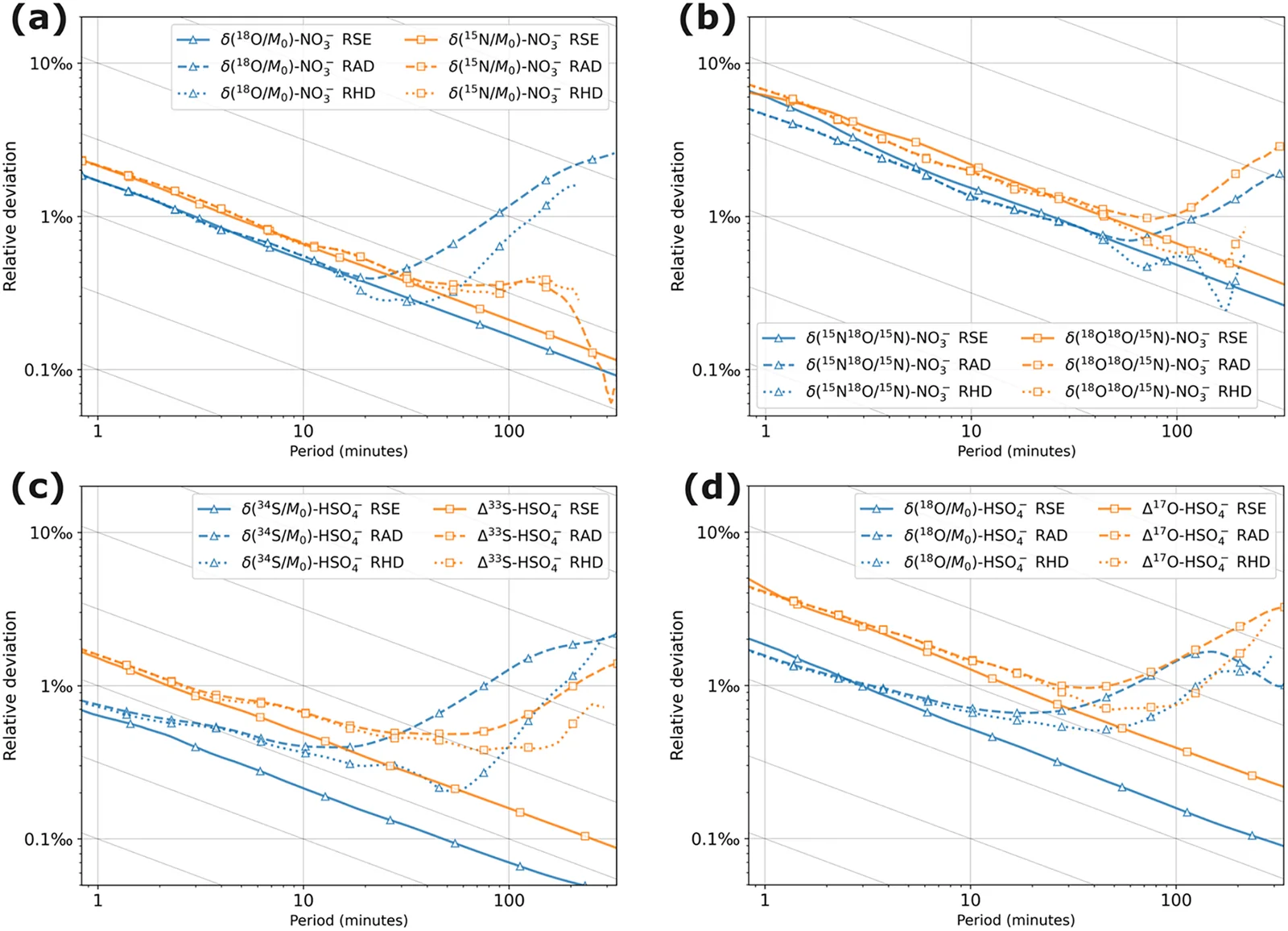
Relative standard error (RSE, solid lines), relative Allan deviation (RAD, dashed lines), and relative Hadamard deviation (RHD, dotted lines) for isotopologue ratios in (a) NO_3_^–^ *M*_0_, (b) NO_3_^–^ *no-M*_0_, and (c, d) HSO_4_^–^ *M*_0_ ESI-Orbitrap-MS experiments, obtained from 12 h infusions of pure solutions by syringe. Uncertainties are within the plotted line widths.

**Table 4:** A Summary of the Stability Metrics Used Here and in Recent Studies^a^

metric	relative standard error (RSE)	relative Allan deviation (RAD)	relative Hadamard deviation (RHD)
**Calculation**	σ/ $\sqrt{n}$ of the *n* scans in period 0 ≤ *t* < τ	RMS first differences between consecutive averaged blocks of length τ	RMS second differences between consecutive averaged blocks of length τ
**Drift-sensitivity**	Not sensitive to drift whose amplitude ≪ interscan scatter	Sensitive to all drift	Sensitive only to nonlinear drift

a Each metric is a function of period, τ, and is calculated from an extended continuous ESI-Orbitrap-MS acquisition. RMS = root-mean-square.

As evident in Figure 5a–d, all three metrics approximately follow shot-noise limits for τ < 20 min for all NO_3_^–^ and HSO_4_^–^ isotopologue ratios. Deflections of RAD and RHD from the $\sqrt{n}$ regime respectively indicate the time scales over which total drift and nonlinear drift deteriorate the precision of isotopologue ratio measurements. ESI-Orbitrap-MS drift is never captured by RSE, rendering it unsuitable as a precision metric. For all NO_3_^–^ and HSO_4_^–^ δ-values, RAD minima indicate that total drift becomes apparent for approximately τ > 20 min, while RHD minima show that nonlinear δ-value drift components take effect only over longer time scales, approximately τ > 30 min. The earlier RAD and RHD minima for δ^34^S-SO_4_^2–^ and δ^18^O–SO_4_^2–^, relative to Δ^33^S-SO_4_^2–^ and Δ^17^O–SO_4_^2–^ in Figure 5c,d imply stronger drift for δ-values than derived triple isotope Δ-values, suggesting that a substantial component of drift arises from mass-dependent fractionation processes. Because bracketed ESI-Orbitrap-MS measurements tolerate linear–but not nonlinear–drift, we conclude that RHD is a more suitable ESI-Orbitrap-MS precision metric than RAD or RSE, and that bracketing should be performed on <30 min time scales. Longer acquisition times would not deliver increased precision because of nonlinear instrumental drift. Moreover, the separation of linear and nonlinear drift components using RHD shows that instrumental drift has less impact on bracketed ESI-Orbitrap-MS measurement precision than implied by RAD alone.

Additionally, Figure 6 shows the correlations between Orbitrap analyzer temperature (measured during analysis by a sensor mounted on the Orbitrap block) and isotopologue ratios of NO_3_^–^ and SO_4_^2–^ reference materials measured during an acquisition sequence. Orbitrap analyzer temperature varied in parallel with ambient temperature, which varied diurnally within a 1 °C range in our laboratory. For both analytes, periods during which isotopologue ratio drifts by 5–10‰ were typically associated with temperature swings of amplitude 0.3–0.5 °C and different behaviors were apparent in different temperature ranges. For NO_3_^–^, no correlations between isotopologue ratio and temperature were apparent for *T* < 34 °C, while weak positive correlations were found for ^17^O and ^18^O ratios at *T* > 34 °C. The ^15^N ratio did not correlate with temperature. For HSO_4_^–^, strong negative correlations between isotopologue ratio and temperature were observed for ^33^S, ^34^S and ^18^O, for *T* < 34.8 °C, with mass-dependent correlation slopes. For *T* > 34.8 °C, weak positive correlations were observed for ^33^S, ^34^S and ^18^O. For HSO_4_^–^, ^17^O variability was noisy and somewhat decoupled from more abundant isotopologues. We infer that, in certain temperature regimes, ESI-Orbitrap-MS isotopologue ratio variability is partially driven by mass-dependent, thermal drift, with a greater thermal dependence for HSO_4_^–^ compared to NO_3_^–^. Possible mass-independent drift of low abundance isotopologue ratios should be investigated further. No corrections of the temperature change effect were made. While we currently cannot explain the distinct behaviors observed across different temperature ranges, plausible sources of the temperature-dependent isotopic drift include the ESI process, ion transmission, and isotopologue detection in the Orbitrap analyzer. Temperature control could reduce ESI-Orbitrap-MS isotopologue ratio drift and perhaps permit longer acquisition durations without penalising precision. For existing instrumentation, this could be achieved by isolating the instrument in an air-conditioned environment, whereas a long-term solution could involve temperature-controlled ESI-Orbitrap-MS instrumentation.

**Figure 6:**
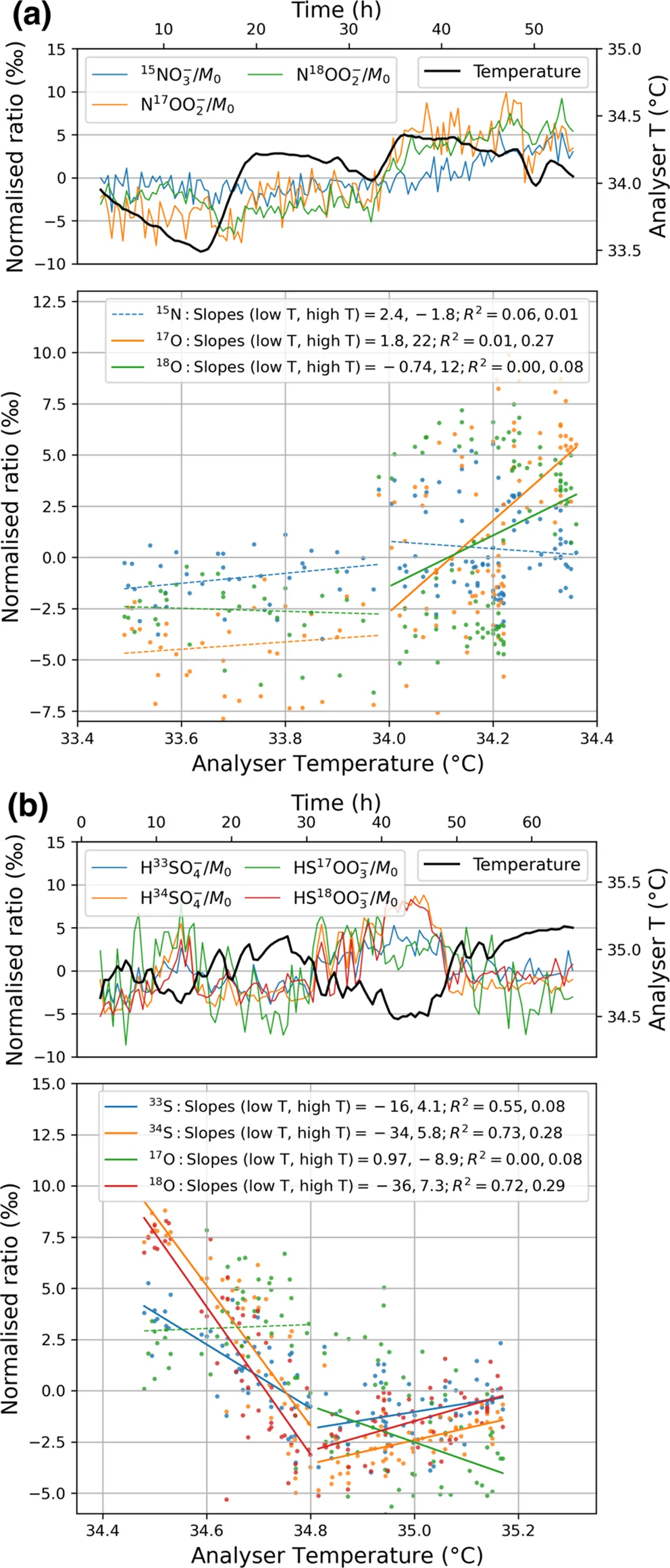
Plots showing ESI-Orbitrap-MS drift for (a) NO_3_^–^ and (b) HSO_4_^–^ isotopologue ratios measured for repeated autosampler injections of the same solutions over approximately 60 h with correlations observed between Orbitrap analyzer temperature and measured isotopologue ratios. Solid and dashed regression lines represent correlations with *p* < 0.05 and *p* > 0.05, respectively, with the temperature domain split at *T* = 34 °C for NO_3_^–^ and *T* = 34.8 °C for HSO_4_^–^.

### Nitrate Isotope Method Intercomparison

ESI-Orbitrap-MS singly substituted NO_3_^–^ isotopologue δ^15^N-NO_3_^–^, δ^18^O-NO_3_^–^, and Δ^17^O-NO_3_^–^ and corresponding denitrifier IRMS elemental isotope measurements^11^ made on the same Mont Blanc ice core samples were used to validate ESI-Orbitrap-MS performance for NO_3_^–^ isotopologues. Such a comparison is justified because isotopic substitutions occur in only around 1% of NO_3_^–^ molecules, and singly substituted isotopologue δ-values remain within 0.1‰ of elemental δ-values even when isotope clumping reaches 10‰.^31^ The intercomparison, illustrated by Bland-Altman plots in Figure 7 and summarized in Table 5, shows that, relative to IRMS, ESI-Orbitrap-MS has 2 times stronger precision for δ^18^O-NO_3_^–^, yet 2 times weaker precision for both δ^15^N-NO_3_^–^and Δ^17^O-NO_3_^–^. The combination of *M*_0_ *and no-M*_0_ data notably improved Δ^17^O-NO_3_^–^ precision by a factor of 1.4 relative to *M*_0_ data alone. In terms of isotopic accuracy, δ^15^N-NO_3_^–^, δ^18^O-NO_3_^–^, and Δ^17^O-NO_3_^–^ all show strong agreement (Concordance Correlation Coefficient (CCC) > 0.78) between ESI-Orbitrap-MS and IRMS, with no large mean biases. The horizontal dashed lines in Figure 7 show that ESI-Orbitrap-MS agrees with IRMS within ±3‰, ±6‰, and ±2‰ (95% limits of agreement) for δ^15^N-NO_3_^–^, δ^18^O-NO_3_^–^, and Δ^17^O-NO_3_^–^, respectively. These results extend the findings of Walters et al. (2026) to include ice core samples while reducing the proportional δ^15^N-NO_3_^–^ bias (gradients in Figure 7) from 0.3 to 0.2 and reducing the mean δ^18^O-NO_3_^–^ bias from 3.3‰ to 1.0‰ (ESI-Orbitrap-MS < IRMS). These accuracy improvements are likely attributable to the comprehensive calibration procedure used in this work and the combination of *M*_0_ and *no-M*_0_ data, while the relatively wide δ^18^O-NO_3_^–^ limits of agreement observed in both studies are suspected to arise from IRMS method uncertainties. By avoiding chemical conversions and potential isotopic fractionations or exchanges,^24,25^ ESI-Orbitrap-MS may provide more reliably accurate δ^18^O-NO_3_^–^ measurements than denitrifier IRMS: this possibility should be investigated by future studies. Line plots showcasing the comparison are presented in Supporting Information Figures S9–S14 and the ESI-Orbitrap-MS and IRMS data are tabulated in Supporting Information Data Set S1. Note that only ten samples had δ^18^O-NO_3_^–^ IRMS measurements and O isotope δ-values for one batch of 11 ESI-Orbitrap-MS samples were discarded because of an erroneous calibration.

**Figure 7:**
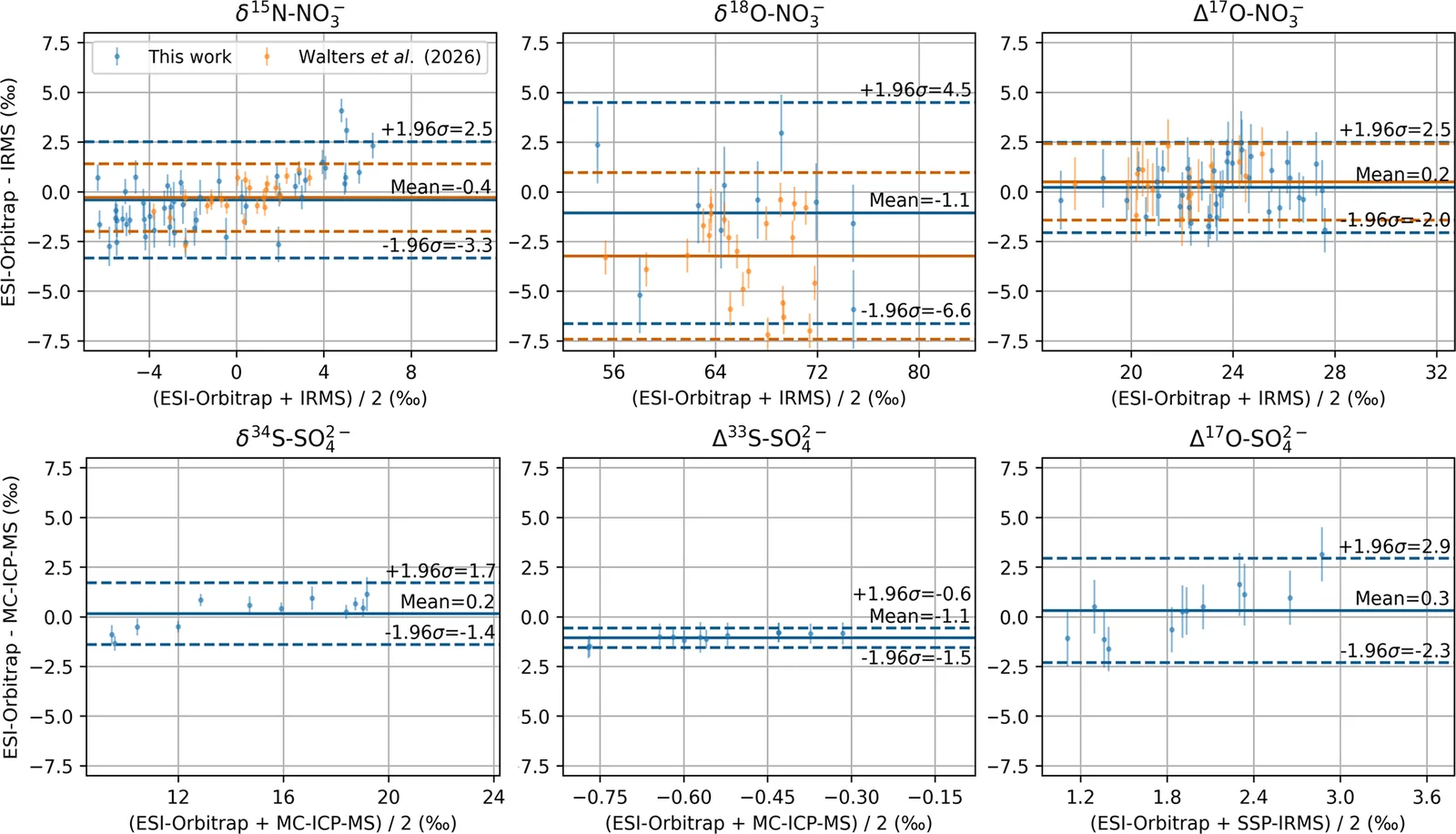
Bland-Altman plots illustrating the NO_3_^–^ ESI-Orbitrap-MS–denitrifier IRMS comparisons of this work (ice core samples) and Walters et al. (2026) (aerosol samples), and the SO_4_^2–^ ESI-Orbitrap-MS–MC-ICP-MS/SSP-IRMS comparison of this work (aerosol samples). Axis labels refer to the average and difference between measurements made using the indicated methods. Vertical bars represent 1σ analytical uncertainties combined between methods; dashed horizontal lines represent 95% limits of agreement; numbers refer to this study only.

**Table 5:** Inter-Method ESI-Orbitrap-MS–Denitrifier IRMS–MC-ICP-MS–SSP-IRMS Comparison for the Ice Core NO_3_^–^ and Aerosol SO_4_^2–^ Samples Measured in This Study^a^

	injected sample amount (nmol)				pooled uncertainty (1σ, ‰)			
NO_3_^–^	orbitrap NO_3_^–^ (*M*_0_ *+no-M*_0_)	denitrifier IRMS		# samples compared	orbitrap NO_3_^–^ (*M*_0_ *+no-M*_0_)	denitrifier IRMS	CCC (95% C.I.)	
δ^15^N	20	100		50	0.6	0.3		0.91 (0.89–0.93)
δ^18^O				10	0.6	1.7		0.87 (0.78–0.94)
Δ^17^O				39	1.0	0.5		0.78 (0.69–0.86)
Δ^15^N^18^O		-		-	2.3	-		-
Δ^18^O^18^O		-		-	3.3	-		-

SO_4_^2–^	orbitrap HSO_4_^–^ (*M*_0_)	ICP-MS	SSP-IRMS	# samples compared	orbitrap HSO_4_^–^ (*M*_0_)	ICP-MS	SSP-IRMS	
δ^34^S	10	400 (5*)	-	12	0.4	0.1	-	0.97 (0.95–0.98)
Δ^33^S				12	0.5	0.2	-	†
δ^18^O		-		-	1.2	-	-	-
Δ^17^O			1000	12	1.3	-	0.2	†

a Inter-method concordance correlation coefficients (CCC) and their confidence intervals (C.I.) were calculated from measurements and measurement uncertainties using a Monte Carlo approach. No comparison was possible for δ^18^O–SO_4_^2–^ due to a lack of IRMS data. * 5–10 nmol SO_4_^2–^ samples are typically required for MC-ICP-MS,^52^ although 400 nmol were used for these samples. † CCC are not meaningful for Δ^33^S-SO_4_^2–^and Δ^17^O–SO_4_^2–^, because samples have only a narrow range of Δ-values.

### Sulfate Isotope Method Intercomparison

Likewise, ESI-Orbitrap-MS singly substituted HSO_4_^–^ isotopologue δ^34^S-SO_4_^2–^, Δ^33^S-SO_4_^2–^, δ^18^O–SO_4_^2–^ and Δ^17^O–SO_4_^2–^ and corresponding MC-ICP-MS, and SSP-IRMS elemental isotope measurements^9,21,53^ made on the same Antarctic aerosol samples were used to validate ESI-Orbitrap-MS performance for SO_4_^2–^ isotopologues. As with NO_3_^–^, singly substituted HSO_4_^–^ isotopologue δ-values are expected to match elemental δ-values within approximately 0.1‰, justifying this comparison. For these samples, no δ^18^O–SO_4_^2**–**^ IRMS measurements were available for comparison. Moreover, SO_4_^2–^ ESI-Orbitrap-MS *no-M*_0_ analyses are not reported for these samples, due to the strong matrix effects discussed in Matrix Effects. The intercomparison, illustrated by Bland-Altman plots in Figure 7 and summarized in Table 5, shows that, despite exhibiting 4 times lower precision than MC-ICP-MS, ESI-Orbitrap-MS δ^34^S-SO_4_^2–^ measurements are accurate (CCC = 0.97), display no large bias, and agree with MC-ICP-MS within ±1.7‰ (95% limits of agreement). Although these samples cover only small ranges in Δ^33^S-SO_4_^2–^ and Δ^17^O–SO_4_^2–^, it appears that Δ^17^O–SO_4_^2–^ is measured accurately by ESI-Orbitrap-MS, with no large bias and agreement with IRMS within ±3‰ (95% limits of agreement), while ESI-Orbitrap-MS Δ^33^S-SO_4_^2–^ measurements exhibit a consistent systematic bias of (−1.1 ± 0.4)‰ (95% limits of agreement). The Δ^33^S-SO_4_^2–^ bias persisted despite isotopic calibration, could be the result of an unresolved type 3 interference in these samples (see Matrix Effects section). Further tests should verify whether this offset is a persistent feature of ESI-Orbitrap-MS Δ^33^S-SO_4_^2–^ analysis. Nonzero gradients in Figure 7 suggest possible proportional biases for δ^34^S-SO_4_^2–^ (slope = 0.17 ± 0.04 (1σ); *p* < 0.05) and Δ^17^O–SO_4_^2–^ (slope = 0.9 ± 0.6 (1σ); p = 0.13). While the limited number of SO_4_^2–^ samples analyzed in this study means that these proportional biases should be interpreted with caution, they may indicate inadequate correction for isotope scale effects during calibration. The demonstrated accuracy of ESI-Orbitrap-MS δ^34^S-SO_4_^2–^ measurements is sufficient for meaningful geochemical interpretations in terrestrial samples, while further accuracy and precision improvements may be required for certain Δ^33^S-SO_4_^2–^ and Δ^17^O–SO_4_^2–^ applications. As observed for NO_3_^–^, successful application of *no-M*_0_ analysis to SO_4_^2–^ samples should enhance single-substituted isotopologue precision, most notably Δ^17^O–SO_4_^2–^, whose measurement precision is currently strongly limited by counting statistics due to the low HS^17^O_4_^–^ abundance in *M*_0_ acquisitions (0.15%). Line plots showcasing the comparison are presented in Supporting Information Figures S9–S14 and the ESI-Orbitrap-MS, MC-ICP-MS and SSP-IRMS data are tabulated in Supporting Information Data Set S1.

The ^2^HSO_4_^–^ and HS^17^OO_3_^–^ isotopologues differ in *m*/*z* by only 20 ppm, requiring mass resolution *m*/δ*m* > 10^5^ to baseline resolve their spectral peaks. In this work, the need for higher mass resolution was avoided by the reasonable assumption that all H atoms in HSO_4_^–^ ions produced from acidified methanolic samples with constant solvent composition have the same ^2^H/^1^H ratio. Under this assumption, interference between ^2^HSO_4_^–^ and HS^17^OO_3_^–^ peaks leads only to a δ-scale compression, which is corrected by calibration. This assumption is likely to also hold for HSO_4_^–^ ions produced from sulfate solutions prepared from a single batch of any protic solvent, since the H atom originates from the solvent H pool during ESI and is not native to any particular sulfate molecule. However, although we find ESI-Orbitrap-MS Δ^17^O–SO_4_^2–^ to be accurate with respect to SSP-IRMS, it remains unclear whether Δ^17^O–SO_4_^2–^ agreement would be maintained at higher analytical precision. An alternative approach avoids this interference altogether by controlled online fragmentation of HSO_4_^–^ and analysis of SO_3_^–^ fragments. Such fragmentation may also remove problematic matrix interferences and allows a lower mass resolution of *m*/δ*m* = 3 × 10^4^ to be used, likely improving isotopologue ratio precision.

### Nitrate Clumped Isotopes in Ice Core Samples

The NO_3_^–^ clumped isotope signatures Δ^15^N^18^O-NO_3_^–^ and Δ^18^O^18^O-NO_3_^–^ were also measured in Mont Blanc ice core samples using ESI-Orbitrap-MS. These provisional clumped isotope Δ-values are calculated relative to existing nitrate isotope standards as described in the Experimental Section and are presented in Figure 8. Pooled Δ^15^N^18^O-NO_3_^–^ and Δ^18^O^18^O-NO_3_^–^ uncertainties were ±2.3‰ and ±3.3‰, while the means and standard deviations of the 14 measurements were (1.0 ± 2.4)‰ and (−1.6 ± 2.3)‰, respectively, suggesting that any intersample clumped isotope variability had similar or smaller amplitude than our analytical uncertainties. The uncertainties–somewhat larger than those reported by Walters et al. (2026)–combine the SEM of six replicate measurements with calibration-associated uncertainty (see Experimental Section). Although interpretations of these ice core nitrate clumped isotope data are beyond the scope of this study, we note that Δ^15^N^18^O-NO_3_^–^ and Δ^18^O^18^O-NO_3_^–^ show no significant (*p* < 0.05) correlations with any single-substituted isotopologue δ-value. In contrast, recent measurements of size-resolved aerosol samples suggested significant correlations between Δ^15^N^18^O-NO_3_^–^, Δ^17^O^18^O-NO_3_^–^ and δ^18^O-NO_3_^–^.^26^ While a direct comparison is impossible because ice cores do not differentiate by particle size, the multipoint calibration protocol used in this work, along with the assumption of intramolecular stochasticity in USGS32, USGS34 and USGS35, may have corrected double-substituted δ-value scale distortions which could otherwise have generated artificial correlations between single-substituted isotopologue δ-values and clumped isotope Δ-values. Robust identification and quantification of such correlations, and interpretation of nitrate clumped isotope variability in natural samples require multipoint δ-value calibrations with consistently reported uncertainties and absolute clumped isotope quantifications of nitrate reference materials. While the former is addressed in this work, the latter should be prioritised in future studies. Moreover, the successful performance of *no-M*_0_ analyses on SO_4_^2–^ samples should allow similar quantification of SO_4_^2–^ clumped isotopes.

**Figure 8:**
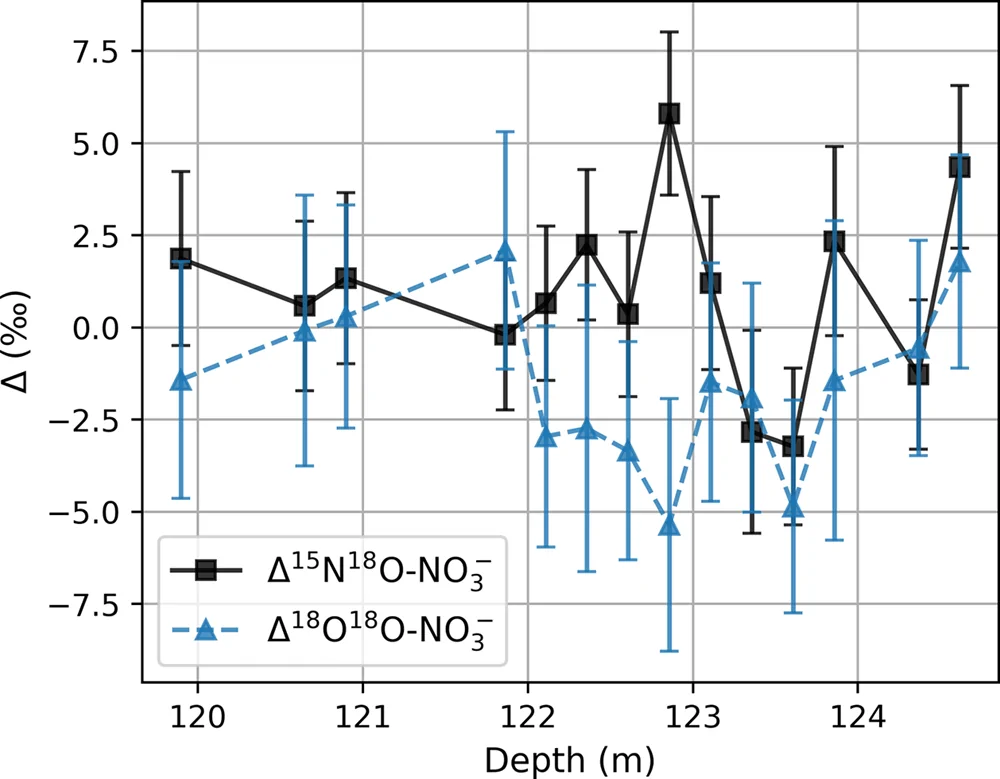
Provisional, relative clumped isotope Δ^15^N^18^O-NO_3_^–^ and Δ^18^O^18^O-NO_3_^–^ values versus depth in Mont Blanc ice core samples, measured by ESI-Orbitrap-MS.

## Conclusions

This work presents and validates a method for multi-isotopologue analysis of both NO_3_^–^ and SO_4_^2–^ in natural samples by ESI-Orbitrap-MS, demonstrating high-precision (±0.4–1.3‰) measurements of δ^15^N-NO_3_^–^, δ^18^O-NO_3_^–^, Δ^17^O-NO_3_^–^, δ^34^S-SO_4_^2–^, Δ^33^S-SO_4_^2–^, δ^18^O–SO_4_^2–^ and Δ^17^O–SO_4_^2–^ in ice core and aerosol samples, and the first simultaneous measurements of S and O triple isotopes in natural SO_4_^2–^ samples. The application of a Monte Carlo multipoint calibration scheme and the combination of *M*_0_ with *no-M*_0_ data gave accurate isotope measurements relative to traditional IRMS and MC-ICP-MS methods, providing the first validation of ESI-Orbitrap-MS for SO_4_^2–^ isotopologues in natural samples and improving the current accuracy for NO_3_^–^ isotopologues in natural samples, while quantifying calibration-associated uncertainties. Relative to existing nitrate isotope standards, we have provisionally quantified Δ^15^N^18^O-NO_3_^–^ and Δ^18^O^18^O-NO_3_^–^ clumped isotope values in ice core samples with analytical uncertainties of several permil: no significant correlations between nitrate clumped isotopes and singly substituted isotopologue δ-values were observed. To enable robust interpretation of the newly accessible clumped isotope information provided by ESI-Orbitrap-MS, future work should improve the precision of and establish absolute reference scales for oxyanion clumped isotope measurements. In contrast to NO_3_^–^, reliable SO_4_^2–^ clumped isotope analyses were compromised by prohibitive matrix interferences during *no-M*_0_ analysis of certain aerosol samples. We found that contaminant peaks with the same integer *m*/*z* as analyte isotopologues, either when baseline resolved and representing as little as 1% of TIC or when nonbaseline-resolved and at similar amplitudes to spectral noise, strongly skewed rare isotopologue measurements. This reveals the strong, previously unreported sensitivity of ESI-Orbitrap-MS isotopologue analyses–especially *no-M*_0_ analyses–to very dilute, near-isobaric, nontarget compounds in natural sample matrices, demonstrating the enhanced necessity of rigorous upstream sample purification and contamination control for ESI-Orbitrap-MS analyses relative to traditional isotopic techniques. Moreover, we show that temperature-dependent ESI-Orbitrap-MS drift can potentially degrade δ-value measurement precision and suggest that users should be wary of memory effects for weakly soluble analytes such as SO_4_^2–^. By sharing such observations, this study advances the isotopic application of ESI-Orbitrap-MS while providing a realistic assessment of its current capabilities and constraints, and guidelines for its application in real-world conditions.

For sufficiently purified samples, our ESI-Orbitrap-MS measurements show no evidence for oxygen isotope artifacts akin to those sometimes observed in nitrate and sulfate IRMS analyses. We speculate that this reflects the absence of chemical conversion steps in the ESI-Orbitrap-MS workflow, which can introduce isotope fractionation or exchange in IRMS-based methods. Although ESI-Orbitrap-MS is certainly susceptible to matrix interferences, it may be less prone to genuine isotope effects than traditional conversion-based methods. Moreover, we demonstrated simultaneous S and O triple isotope measurements of SO_4_^2–^ using ESI-Orbitrap-MS with injected sample amounts of only 10 nmol, substantially simplifying the analytical workflow and reducing sample requirements compared to the combined MC-ICP-MS and IRMS methods traditionally required to obtain equivalent isotopic information. Although ESI-Orbitrap-MS itself requires only minimal sample, the overall workflow sensitivity is currently limited by the IC purification step, for which 100 nmol samples were targeted. This workflow sample requirement represents an order-of-magnitude reduction relative to the combined MC-ICP-MS and IRMS workflow for S and O isotope analysis of SO_4_^2–^ but is comparable to denitrifier IRMS for N and O isotopes of NO_3_^–^. Improvements to IC sensitivity and upstream blank levels are needed for true nanomole oxyanion isotopologue measurements in natural samples and would facilitate multi-isotopologue and clumped isotope research on small, dilute, and precious samples, including paleoclimate archives and extraterrestrial materials.

Despite several advances, some instrumental limitations will be difficult to overcome. Because the ESI-Orbitrap-MS ion-counting rate is physically limited, existing ESI-Orbitrap-MS instrumentation cannot currently match the <0.2‰ isotopic precision offered by IRMS or MC-ICP-MS while retaining reasonable acquisition times–especially for Δ^33^S-SO_4_^2–^ and Δ^17^O–SO_4_^2–^–and it remains to be verified whether ESI-Orbitrap-MS would achieve subpermil accuracy relative to IRMS for these isotope systems if more replicate injections were performed and averaged. Moreover, although we successfully mitigated matrix effects through sample cleanup using single-stage IC and by ensuring identical treatment between samples and isotope standards, our findings suggest that applying ESI-Orbitrap-MS to different analytes, more complex sample matrices or higher-precision isotopic analyses may require enhanced sample cleanup.

Although it does not yet rival the isotopic precision of IRMS or MC-ICP-MS techniques, the ESI-Orbitrap-MS approach accesses previously inaccessible NO_3_^–^ clumped isotope dimensions and facilitates SO_4_^2–^ multi-isotope analysis on nanomole-size samples while being readily extensible to other nonvolatile solutes and sample matrices. The potential of ESI-Orbitrap-MS to deliver accurate compound-specific, position-specific and clumped isotope data across diverse analytes and sample types represents a long-awaited advance in analytical science, the full scope of which will emerge as further studies explore its capabilities.

## Supplementary Material





## Data Availability

The data treatment scripts used in this work are archived on Zenodo (https://doi.org/10.5281/zenodo.19495113)^58^ and comprise the following four Jupyter Notebooks, each containing Python code. *Extract-Isotopologue-Orbitrap.ipynb* converts a sequence of *.Isox* data files to internal δ-values, see Experimental Section. *Calibrate-Orbitrap-NO3.ipynb* Converts internal δ-values to calibrated δ-values, see Experimental Section and Supporting Information Text S3. *Calibrate-Orbitrap-SO4-M0.ipynb* converts internal δ-values to calibrated δ-values, see Experimental Section and Supporting Information Text S3. *Calculate-M0 + noM0-Orbitrap-NO3.ipynb* combines δ-values from M_0_ and *no-M_0_* acquisitions, see Supporting Information Text S4. and the data supporting the findings of this study are archived on the PANGAEA database, comprising the following data sets: ESI-Orbitrap-MS *.RAW* files and *.Isox* files containing raw data from continuous NO_3_^–^ and HSO_4_^–^ acquisitions used for calculation of RSE, RAD, and RHD (https://doi.pangaea.de/10.1594/PANGAEA.990372 ^55^). ESI-Orbitrap-MS.*RAW* files, *.Isox* files, injection sequence *.csv* files, and *.xlsx* sheets containing raw mass spectral data and treated isotopologue data for Mont Blanc ice core NO_3_^–^ samples. (https://doi.pangaea.de/10.1594/PANGAEA.990363 ^56^) ESI-Orbitrap-MS.*RAW* files, *.Isox* files, injection sequence *.csv* files, and *.xlsx* sheets containing raw mass spectral data and treated isotopologue data for Antarctic aerosol SO_4_^2–^ samples. (https://doi.pangaea.de/10.1594/PANGAEA.990367 ^57^).

## Notes

ESI-Orbitrap-MS – IRMS – MC-ICP-MS method comparison (Data set S1) (XLSX)
ESI-Orbitrap-MS acquisition parameters and observations (Table S1); NO_3_^–^ and HSO_4_^–^ isotopologue masses and abundances (Table S2); determination of ESI-Orbitrap-MS isotopic memory effect (Text S1); ESI-Orbitrap-MS isotopic memory effect tests (Figures S1–S2); sources and isotopic characterization of new Na_2_SO_4_ isotope standard materials (Text S2); isotope values of new Na_2_SO_4_ isotope standard materials (Table S3); ESI-Orbitrap-MS δ-value calibration details (Text S3); ESI-Orbitrap-MS δ-value calibrations (Figures S3–S5); protocol for combining ESI-Orbitrap-MS NO_3_^–^ *M*_0_ and *no-M*_0_ data (Text S4); interferences in ESI-Orbitrap-MS HSO_4_^–^ *no-M*_0_ analysis (Figure S6); ESI-Orbitrap-MS statistical testing protocol (Text S5); ESI-Orbitrap-MS statistical testing results (Figures S7–S8); line plots for ESI-Orbitrap-MS–IRMS–MC-ICP-MS method comparison (Figures S9–S14) (PDF)
