# Supplementary material for: Electrospray-Orbitrap Analysis of Nitrate and Sulfate Isotopologues in Natural Samples: Performance and Constraints
Source: Anal Chem. 2026 Aug 31;98(36):26323–38. doi: 10.1021/acs.analchem.6c00183 (PMC13584694; doi:10.1021/acs.analchem.6c00183)
Supplement: Supplementary file 1 [file ac6c00183_si_002.pdf]

SUPPORTING INFORMATION

Electrospray-Orbitrap Analysis of Nitrate and Sulphate Isotopologues in Natural Samples: Performance and Constraints

Jack Saville[1], Julien Witwicky[1], Elsa Gautier[1], Alexis Lamothe[1,2], Nils Johannes Kuhlbusch[3,4], Becky Alexander[5], Andrew Schauer[6], Andrea Burke[7], Priyesh Prabhat[7], Eva Stüeken[7], Joshua Garber[7], Nicolas Caillon[1], Joël Savarino[1*].*

AUTHOR ADDRESSES

1. Univ. Grenoble Alpes, CNRS, IRD, Grenoble-INP, INRAE, IGE, Grenoble, 38000, France
2. International Center for Isotope Effects Research, Nanjing University, Nanjing 210023, China
3. University of Münster, 48149 Münster, Germany
4. Thermo Fisher Scientific GmbH, 28199 Bremen, Germany
5. Department of Atmospheric and Climate Sciences, University of Washington, Seattle, Washington, USA
6. Department of Earth and Space Sciences, University of Washington, Seattle, Washington, USA
7. University of St Andrews, School of Earth & Environmental Sciences, St Andrews, KY16 9TS, United Kingdom

CORRESPONDING AUTHORS

* jack.saville@univ-grenoble-alpes.fr

* joel.savarino@cnrs.fr

TABLE OF CONTENTS

ESI-ORBITRAP-MS ACQUISITION PARAMETERS AND OBSERVATIONS

Table S1: ESI-Orbitrap-MS acquisition parameters and observations

NO_3^- AND HSO_4^- ISOTOPOLOGUE MASSES AND ABUNDANCES

Table S2: NO_3^- and HSO_4^- isotopologue masses and abundances

ESI-ORBITRAP-MS ISOTOPIC MEMORY EFFECT

Text S1: Determination of ESI-Orbitrap-MS isotopic memory effect

Figures S1 – S2: ESI-Orbitrap-MS isotopic memory effect tests

NEW Na_2SO_4 ISOTOPE STANDARD MATERIALS

Text S2: Sources and isotopic characterisation of new Na_2SO_4 isotope standard materials

Table S3: Isotope values of new Na_2SO_4 isotope standard materials

ESI-ORBITRAP-MS δ-VALUE CALIBRATON

Text S3: ESI-Orbitrap-MS δ-value calibration details

Figures S3 – S5: ESI-Orbitrap-MS δ-value calibrations

COMBINING ESI-ORBITRAP-MS NO_3^- M_0 AND *no-*M_0 DATA

Text S4: Protocol for combining ESI-Orbitrap-MS NO_3^- M_0 and *no-*M_0 data

INTERFERENCE IN ESI-ORBITRAP-MS HSO_4^- *no-*M_0 ANALYSIS

Figure S6: Interferences in ESI-Orbitrap-MS HSO_4^- *no-*M_0 analysis

ESI-ORBITRAP-MS STATISTICAL TESTING

Text S5: ESI-Orbitrap-MS statistical testing protocol

Figures S7 – S8: ESI-Orbitrap-MS statistical testing results

ESI-ORBITRAP-MS – IRMS – MC-ICP-MS METHOD COMPARISON

Figures S9 – S14: Line plots for ESI-Orbitrap-MS – IRMS – MC-ICP-MS method comparison

Data set S1: ESI-Orbitrap-MS – IRMS – MC-ICP-MS method comparison (separate *.xlsx* file)

ESI-ORBITRAP-MS ACQUISITION PARAMETERS AND OBSERVATIONS

Table S1: Summary of ESI-Orbitrap-MS parameters and observations. Arb: arbitrary gas flow units; ITT: ion transfer tube; RF: radiofrequency ion optics; TIC: total ion current; RSD: relative standard deviation; AGC: automatic gain control; IT: injection time (time taken to accumulate ions for a microscan).

**Electrospray parameter**	**Value or value range**	
Negative spray voltage	2000 – 2200 V	
Sheath gas	1 – 2 Arb. (0.3 – 0.6 L/min)	
Auxiliary gas	1 – 2 Arb. (1.7 – 3.4 L/min)	
Sweep gas	0	
Electrospray heating	None	
ITT temperature	280 °C	
RF voltage	100 %	
**Orbitrap parameter**	**NO_3^- $\boldsymbol{M_0}$ (*no-M_0*)**	**HSO_4^- $\boldsymbol{M_0}$**
Scan type	Full scan	Full scan
Mass resolution (at m/z = 200)	15,000	45,000
AGC target	3×10^5	1×10^6
m/z isolation window	61.2 – 67.0 (62.2 – 68) Da	95.5 – 100.5 Da
Maximum IT	1000 ms	1000 ms
Microscan number	10	10
**Observations with 45 *μ*M analyte**	**NO_3^- $\boldsymbol{M_0}$ (*no-M_0*)**	**HSO_4^- $\boldsymbol{M_0}$**
TIC	100 – 300 (1 – 3) $\times 10^6$ s^{-1}	200 – 500 $\times 10^6$ s^{-1}
TIC RSD	2 – 10 (1 – 4) %	1 – 6 %
Spray current	0.1 – 0.2 μA	0.1 – 0.3 μA
IT	1 – 3 (100 – 300) ms	2 – 5 ms
Microscan duration	36 ms	105 ms
Microscan frequency	28 (3.3 – 10) s^{-1}	9.5 s^{-1}
Orbitrap ion analysis rate	8.4 (1– 3) $\times 10^6$ s^{-1}	9.5 $\times 10^6$ s^{-1}
Isotopologues available	M_0, ^{15}N, ^{17}O, ^{18}O, (^{15}N, ^{17}O, ^{18}O, $^{15}N^{18}O$, $^{18}O^{18}O$)	M_0, ^{33}S, ^{17}O, ^{34}S, ^{18}O

NO_3^- AND HSO_4^- ISOTOPOLOGUE MASSES AND ABUNDANCES

Table S2: NO_3^- and HSO_4^- non-substituted, singly-substituted, and doubly-substituted isotopologue masses and relative abundances in M_0 and no-M_0 experiments, calculated from average N, S and O isotopic abundances. Abundances marked * reflect the isotopologues quantified in this work, in M_0 or no-M_0 acquisitions.

**Substitution(s)**	**Mass (a.m.u.)**	**Relative abundance in M_0 experiment ($\times 10^{-6}$)**	**Relative abundance in no-M_0 experiment ($\times 10^{-6}$)**
		**NO_3^-**	
M_0 (unsubstituted)	61.9884	989,100 *	-
^{15}N	62.9854	3614 *	332,000 *
^{17}O	62.9926	1130 *	103,800 *
^{15}N^{17}O	63.9896	4	367
^{18}O	63.9926	6098 *	560200 *
^{17}O^{17}O	63.9968	0.4	37
^{15}N^{18}O	64.9896	22	2020 *
^{17}O^{18}O	64.9968	5	460
^{18}O^{18}O	65.9969	13	1194 *
		**HSO_4^-**	
M_0 (unsubstituted)	96.9601	940,600 *	-
^{33}S	97.9595	7427 *	125,000
^{17}O	97.9643	1433 *	24,121
^{2}H	97.9664	108	1818
^{34}S	98.9559	42,080 *	708,300
^{33}S^{17}O	98.9637	11	185
^{18}O	98.9643	7732 *	130,200
^{34}S^{17}O	99.9601	64	1077
^{2}H^{34}S	99.9622	5	81
^{33}S^{18}O	99.9637	61	1027
^{17}O^{18}O	99.9686	9	151
^{36}S	100.9551	99	1666
^{348}O	100.9601	346	5824
^{18}O^{18}O	100.9686	24	403

ESI-ORBITRAP-MS ISOTOPIC MEMORY EFFECT

Text S1: Determination of ESI-Orbitap-MS isotopic memory effects

The isotopic effect of a preceding autosampler injection on an ESI-Orbitrap-MS δ-value measurement was quantified by tracking the temporal evolution of the measured isotopologue ratio throughout injections. For autosampler injections in which the mean measured δ-value ($\bar{\delta}_i$= average of ca. 1000 scans) differed from that of the preceding injection ($\bar{\delta}_{i-1}$) by greater than 1 ‰, δ-values were caluclated for each scan (δ_{scan}), relative to the average isotopologue ratio in the preceding autosampler injection. These δ_{scan} values were then divided by the mean δ-value difference between the preceding and current injections, $\bar{\delta}_i - \bar{\delta}_{i-1}$.

$$\phi = \frac{\delta_{scan}}{\bar{\delta}_i - \bar{\delta}_{i-1}} \cong \frac{R_{scan} - \bar{R}_{i-1}}{\bar{R}_i - \bar{R}_{i-1}} \qquad \text{(Equation S1)}$$

This gave, for each injection, a normalised ϕ value per scan, where $\phi = 0$ represented a measured isotopologue ratio equal to the previous injection mean, $\phi = 1$ represented a measured value equal to the current injection mean, while $\phi < 0$ or $\phi > 1$ would represent a measured isotopologue ratio beyond the range of the current and previous injection means. To suppress measurement noise, the ϕ signal was averaged over several hundred injections in a single sequence, revealing the instrumental memory signal beneath. To the stacked signal, a function of the form $\phi(t) = A[1 - \exp(-bt)]$ was fitted, where t represented the time since the end of the previous autosampler injection plateau. This parametrisation represents mixing between the decaying contribution of the preceding injection, $A\exp(-bt)$, and the constant signal from the current injection, A. The most abundant NO_3^- and HSO_4^- isotopologues were found to give the most coherent ϕ signals (see Figures S2a+b). The fit parameter A represented the ϕ value that would have been measured in the absence of instrumental memory effects. Its difference from unity, $M = 1 - \frac{1}{A}$, was averaged over the ^{15}N and ^{18}O NO_3^- isotopologues and over the ^{34}S, ^{33}S and ^{18}O HSO_4^- isotopologues. The different apparent behaviour of ^{17}O-substituted isotopologues compared to more abundant isotopologues was assumed to be related to their low counting statistics and low measurement precisions. For NO_3^-, the averaged M was equal to zero, meaning no instrumental memory effect was detected, while for HSO_4^-, the averaged M was (6 ± 2) %,

and is the quantity cited in the paper's Experimental Section. The following figures present plots of stacked ϕ signals for NO_3^- and HSO_4^- isotopologue ratios.

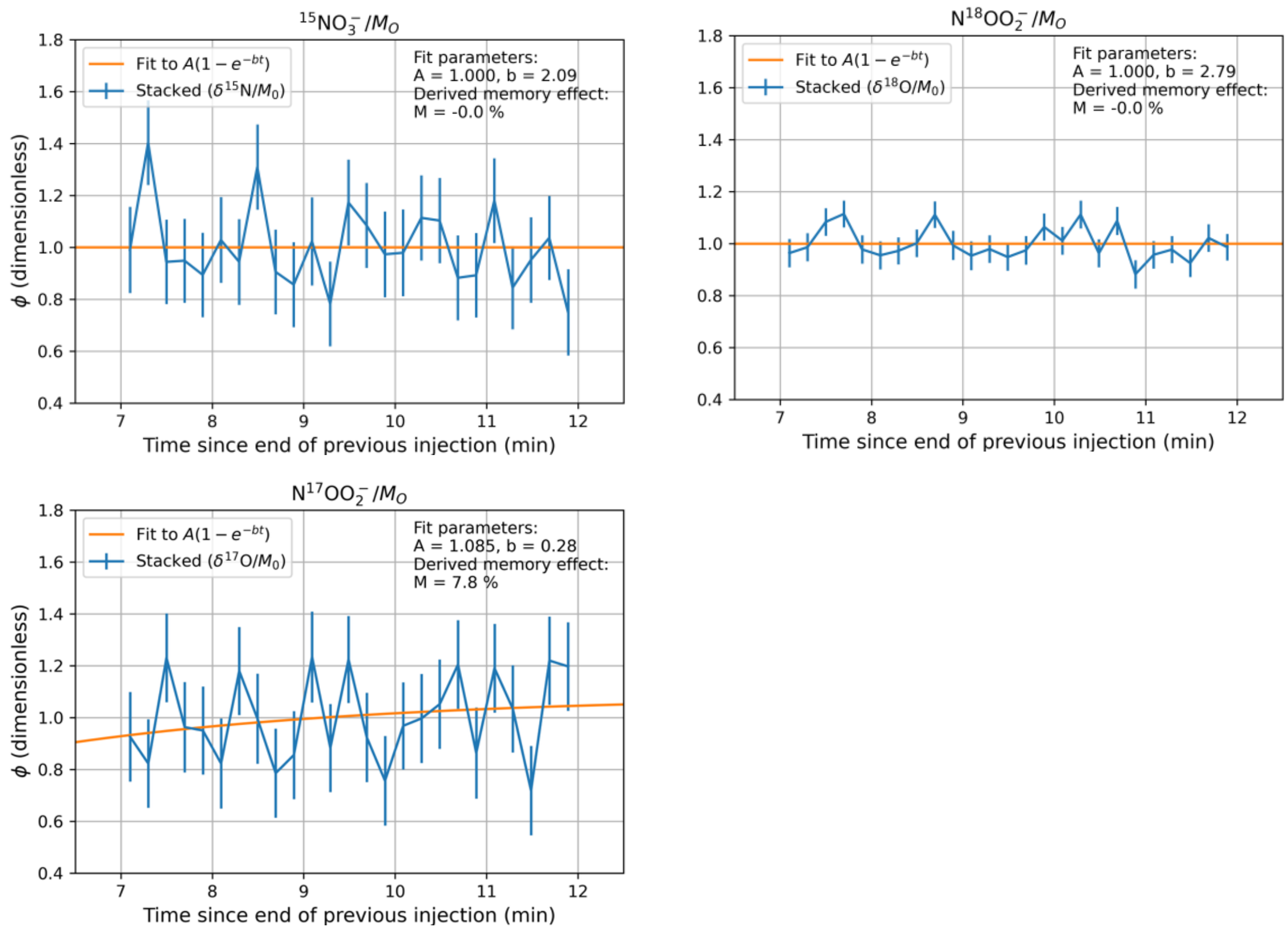

Figure S1: Memory effect testing for NO_3^- isotopologue ratios. Blue error bars represent 1 s.d. of the stacked data.

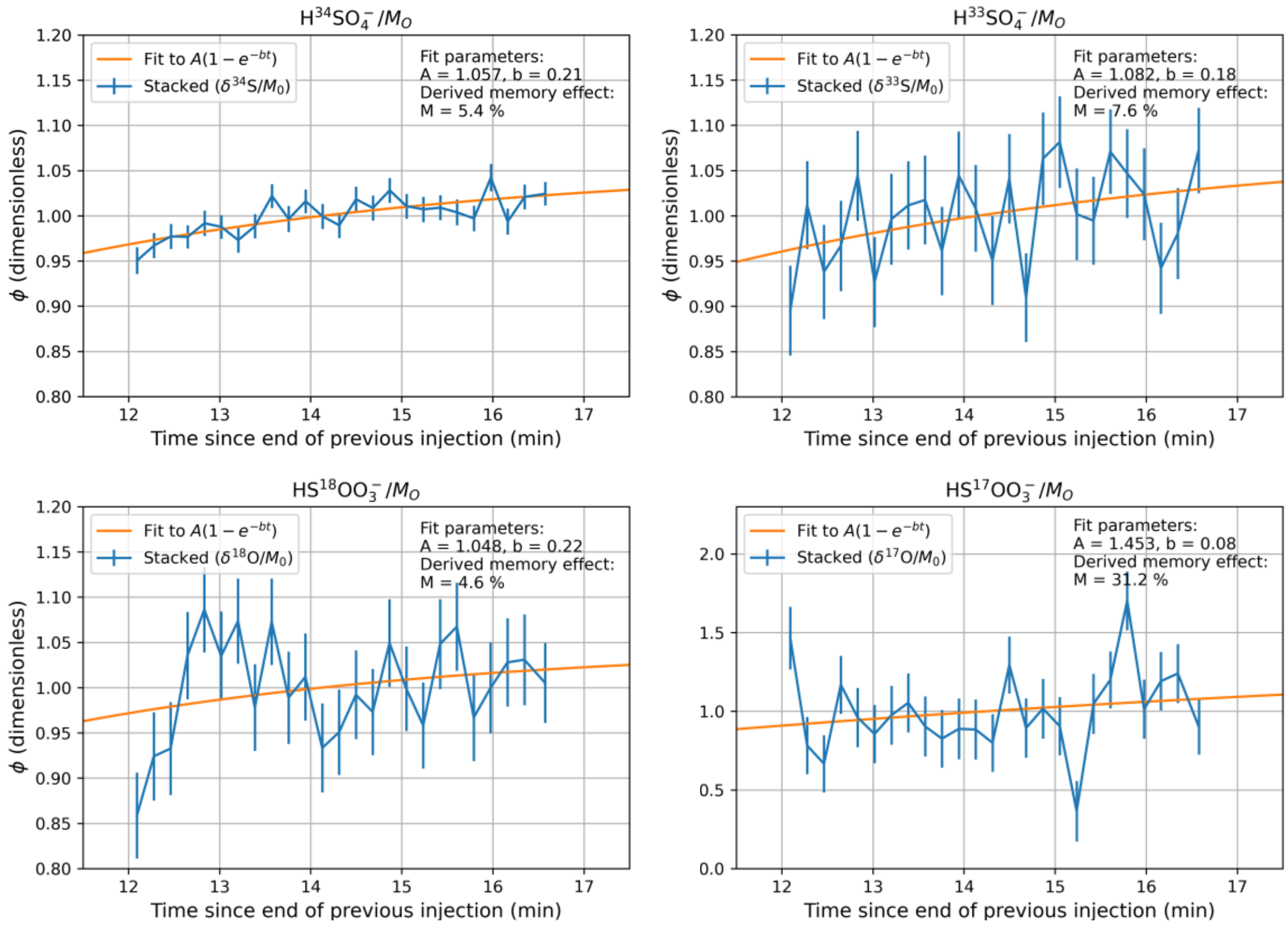

Figure S2: Memory effect testing for HSO_4^- isotopologue ratios. Blue error bars represent 1 s.d. of the stacked data.

NEW Na_2SO_4 ISOTOPE STANDARD MATERIALS

Text S2 Sources and isotopic characterisation of new Na_2SO_4 isotope standard materials

In the paper's Experimental Section and Table 2, we present a series of new Na_2SO_4 isotope standard materials, whose soluble nature makes them useful for ESI-Orbitrap-MS isotopologue analysis. These four isotope standards – SodSul-2, SodSul-4, SodSul-6 and SodSul-8 – cover wide ranges in $\delta^{34}S\text{-}SO_4^{2-}$ and $\delta^{18}O\text{-}SO_4^{2-}$ and possess near-zero $\Delta^{33}S\text{-}SO_4^{2-}$. Here, we present details on their sources and the provisional results of an ongoing interlaboratory comparison project to quantify their $\delta^{34}S\text{-}SO_4^{2-}$, $\delta^{33}S\text{-}SO_4^{2-}$ and $\delta^{18}O\text{-}SO_4^{2-}$.

Sources Several commercially-available Na_2SO_4 materials were purchased, of which SodSul-2, SodSul-4, SodSul-6 and SodSul-8 were identified as isotopically desirable and were distributed. SodSul-2 is a reagent grade anhydrous Na_2SO_4 (Sigma Aldrich, part number 238597) and was baked for 24 hours at 500 ^{O}C, dissolved in ultrapure water, filtered at 8 μm, and freeze dried. SodSul-4 is a reagent grade anhydrous Na_2SO_4 (Fisher Chemical, part number S421-500). SodSul-6 is a commercial Na_2SO_4 (Natural Sodium Sulfate, Duda Energy). SodSul-8 is a thenardite from Soda Lake, Carrizo Plain, San Luis Obispo County, CA, USA and was ground from its original crystalline form to a fine powder using pestle and mortar.

Isotopic characterisation The four Na_2SO_4 standards were distributed to three different laboratories for measurement of $\delta^{34}S\text{-}SO_4^{2-}$ by EA-IRMS following Fry et al. (2002) and Stüeken et al. (2020), $\delta^{34}S\text{-}SO_4^{2-}$, $\delta^{33}S\text{-}SO_4^{2-}$ and $\Delta^{33}S\text{-}SO_4^{2-}$ by MC-ICP-MS following Yu et al. (2025) and $\delta^{18}O\text{-}SO_4^{2-}$ by Elemental Analysis (EA-)IRMS following Kornexl et al. (1999). Isotopic measurements of each standard are tabulated in Table S3. Presently, the $\Delta^{33}S\text{-}SO_4^{2-}$ and $\delta^{18}O\text{-}SO_4^{2-}$ of the standards have each been measured in only one laboratory. The reported $\Delta^{33}S\text{-}SO_4^{2-}$ and $\delta^{18}O\text{-}SO_4^{2-}$ means and standard deviations are used as provisional estimates of the true values of the standards. In contrast, the $\delta^{34}S\text{-}SO_4^{2-}$ of the standards has been measured in four laboratories, using two different techniques. A hierarchical (random-effects) weighted-mean $\delta^{34}S\text{-}SO_4^{2-}$ which accounts for both inter-laboratory and intra-laboratory variance was used to estimate the likely true $\delta^{34}S\text{-}SO_4^{2-}$ range of the standards.

Table S3: Tabulated provisional results of an ongoing interlaboratory comparison project to quantify the $\delta^{34}S\text{-}SO_4^{2-}$, $\delta^{33}S\text{-}SO_4^{2-}$, $\Delta^{33}S\text{-}SO_4^{2-}$ and $\delta^{18}O\text{-}SO_4^{2-}$ of the Na_2SO_4 isotope standards SodSul-2, SodSul-4, SodSul-6 and SodSul-8. UW = University of Washington IsoLab, USA; WSU = Washington State University Stable Isotope Core Facility, USA; STAiG = St Andrew's Isotope Group, UK. Bold values are the provisional best estimates of the isotopic composition of these materials and correspond to the values reported in Table 2 of the paper. The reported average $\delta^{34}S\text{-}SO_4^{2-}$ and its 1σ uncertainty account for both intra-laboratory and inter-laboratory variance. $\delta^{34}S\text{-}SO_4^{2-}$, $\delta^{33}S\text{-}SO_4^{2-}$ and $\Delta^{33}S\text{-}SO_4^{2-}$ are related by the formula in Table 3 of the paper.

**Quantity**	**Method**	**Laboratory**	**Mean value ± standard deviation (‰)**			
			**SodSul-2**	**SodSul-4**	**SodSul-6**	**SodSul-8**
$\delta^{34}S\text{-}SO_4^{2-}$	EA-IRMS	UW	10.49 ± 1.12	0.76 ± 0.97	10.82 ± 0.99	-9.98 ± 0.73
$\delta^{34}S\text{-}SO_4^{2-}$	EA-IRMS	WSU	11.85 ± 0.21	1.42 ± 0.35	11.32 ± 0.65	-8.42 ± 1.06
$\delta^{34}S\text{-}SO_4^{2-}$	EA-IRMS	STAiG	11.31 ± 0.18	1.25 ± 0.18	11.48 ± 0.18	-9.81 ± 0.18
$\delta^{34}S\text{-}SO_4^{2-}$	MC-ICP-MS	STAiG	10.81 ± 0.01	0.70 ± 0.02	11.04 ± 0.03	-9.81±0.03
$\delta^{34}S\text{-}SO_4^{2-}$	Average	-	**11.21 ± 0.46**	**0.88 ± 0.21**	**11.04 ± 0.03**	**-9.81 ± 0.03**
$\delta^{33}S\text{-}SO_4^{2-}$	MC-ICP-MS	STAiG	**5.61 ± 0.03**	**0.40 ± 0.04**	**5.69 ± 0.05**	**-5.03 ± 0.03**
$\Delta^{33}S\text{-}SO_4^{2-}$	MC-ICP-MS	STAiG	**0.05 ± 0.03**	**0.04 ± 0.04**	**0.02 ± 0.05**	**0.03 ± 0.03**
$\delta^{18}O\text{-}SO_4^{2-}$	SSP-IRMS	UW	**7.09 ± 0.38**	**7.55 ± 0.32**	**14.78 ± 0.29**	**11.98 ± 0.47**

ESI-ORBITRAP-MS δ-VALUE CALIBRATON

Text S3 ESI-Orbitrap-MS δ-value calibration details

ESI-Orbitrap-MS isotopologue measurements of isotope standards, prepared in the same batch as a series of natural samples and analysed in the same M_0 or *no*-M_0 sequence, alongside isotope standard reference values, were used to place natural sample measurements on international isotope scales by linear calibration. A Monte-Carlo method with 1000 bootstraps was used to account for calibration uncertainty. With $x = \delta_{measured}$ and $y = \delta_{true}$, the calibration model,

$$y = (x + 1)^b e^a - 1, \qquad \text{(Equation S2)}$$

can be expressed in linear form as follows

$$\ln(y + 1) = b \ln(x + 1) + a. \qquad \text{(Equation S3)}$$

The calibration points corresponding to isotope standards, (X_i, Y_i), were generated by randomly perturbing ESI-Orbitrap-MS internal δ-value measurements and isotope reference values by their measurement uncertainties. Values were randomly sampled from normal distributions determined from the mean and standard error on the mean of replicate ESI-Orbitrap-MS measurements and the corrsponding isotope standard reference value and its associated uncertainty. For isotopologue ratios with multiple isotope standards available for calibration, Equation S3 was used to perform a least-squares linear regression. The slope and intercept of the regression line gave the parameters a and b. ESI-Orbitrap-MS internal δ-value measurements of samples gave values, x_j, and Equation S2, with the calculated parameters a and b, then gave calibrated δ-values, y_j. This calculation was repeated for n = 1, 2, 3, … 1000, randomly resampling $(X_i^{(n)}, Y_i^{(n)})$ each time to yield a 1000-large set of values $y_j^{(n)}$ for each isotopologue ratio whose mean represents the best estimate of the calibrated δ-value and whose standard deviation represents its calibration-associated uncertainty. These statistics were calculated and reported for each available δ-value, for every sample in a sequence.

If only a single isotope standard was available for calibration for a certain isotopologue, the parameter b was fixed and a was calculated from the single calibration point. For $\delta^{15}N\text{-}NO_3^-$

calibrations using isotope standards USGS34 and USGS35, a 1-point calibration was applied, using USGS35 only and $b = 1.1$, to avoid the sizable uncertainty on b induced by the isotopic proximity of the two standards. For this reason, use of an additional standard with a substantially different $\delta^{15}N\text{-}NO_3^-$ such as USGS32, is preferred. Additionally, some isotope standards have an incomplete complement of reference δ-values. For example, several SO_4^{2-} isotope standards have reference $\Delta^{33}S\text{-}SO_4^{2-}$ values without the corresponding $\delta^{33}S\text{-}SO_4^{2-}$ or $\delta^{34}S\text{-}SO_4^{2-}$ values. To account for these cases, the $\delta^{33}S\text{-}SO_4^{2-}$ of each standard was calculated using its calibrated $\delta^{34}S\text{-}SO_4^{2-}$ value (measured and obtained as above) and its reference $\Delta^{33}S\text{-}SO_4^{2-}$ value, following Schoenemann et al. (2013). Once the $\delta^{33}S\text{-}SO_4^{2-}$ values were calculated for the relevant standards, a calibration was performed as described above.

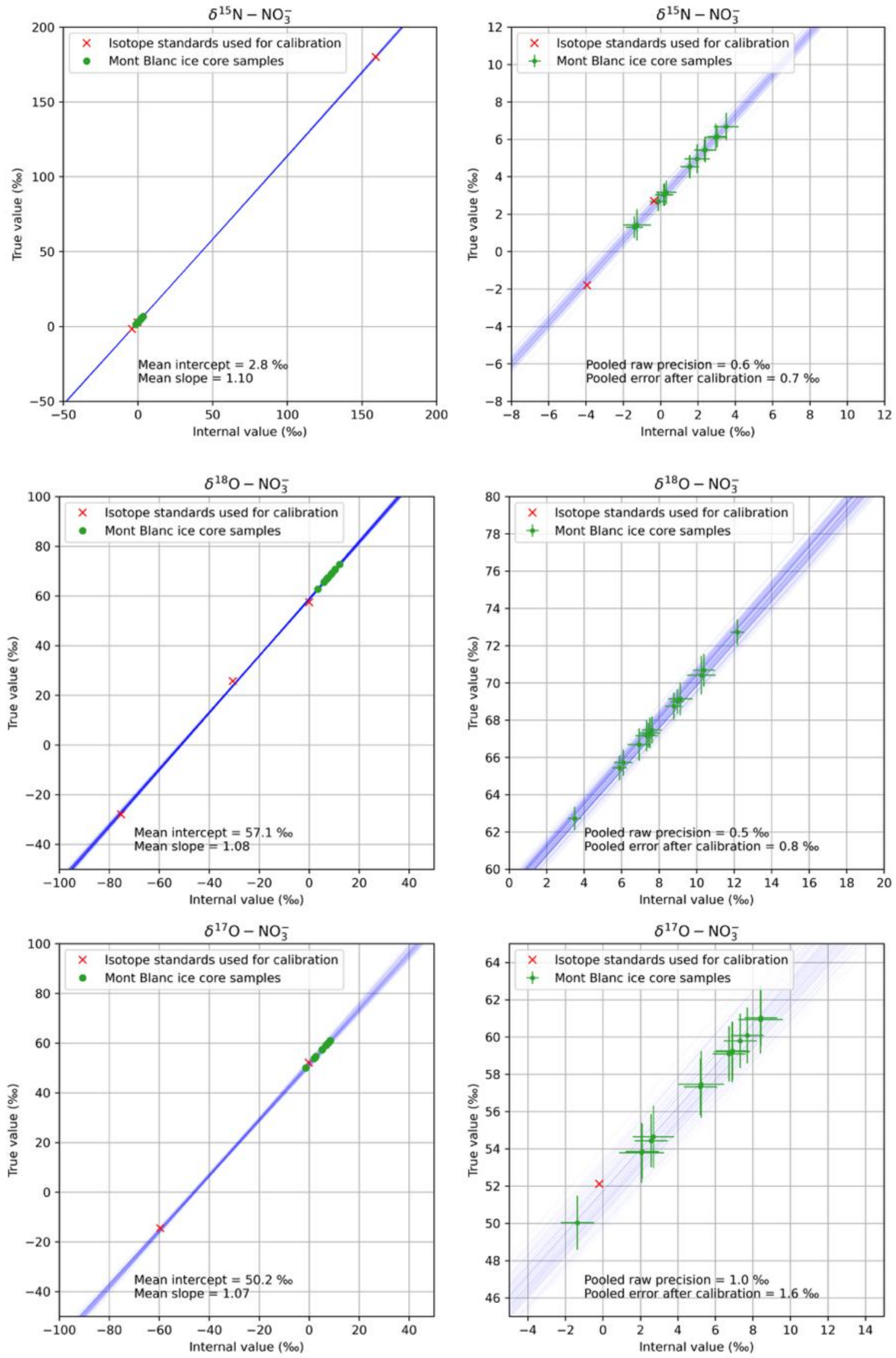

Figure S3: Monte-Carlo linear regressions used for calibration of NO_3^- M_0 ESI-Orbitrap isotopologue δ-values. Right-hand plots are equivalent to left-hand plots except for magnified scales. Calibration standards are indicatd by red crosses, while values of Mont Blanc ice core samples, their raw precisions, σ_{SEM}, and final uncertainties $(\sigma_{SEM}^2 + \sigma_{MC}^2)^{0.5}$, are indicated in green.

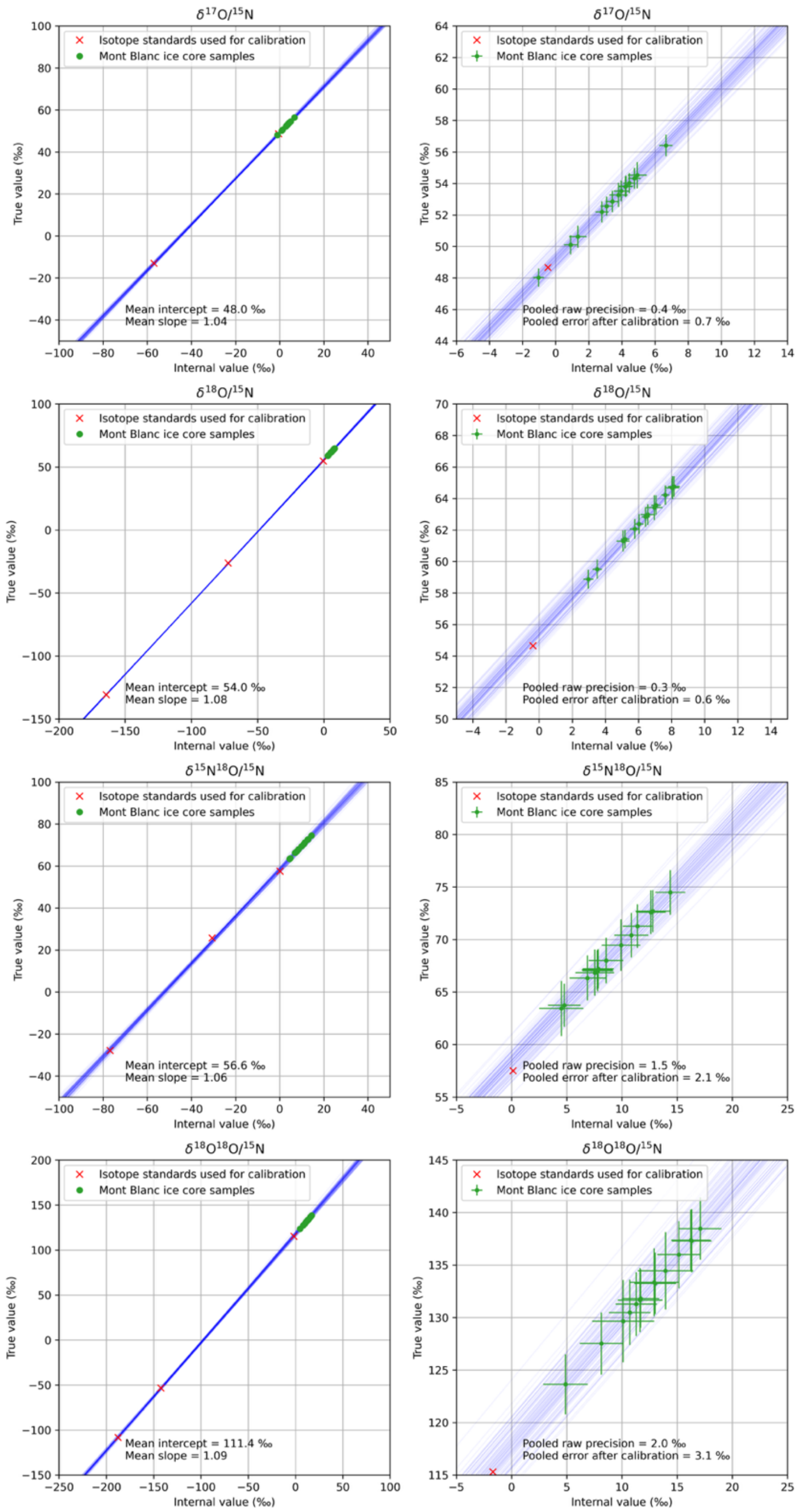

Figure S4: Monte-Carlo linear regressions used for calibration of NO_3^- *no-M₀* ESI-Orbitrap isotopologue δ-values. Right-hand plots are equivalent to left-hand plots except for magnified scales. Calibration standards are indicatd by red crosses, while values of Mont Blanc ice core samples, their raw precisions, σ_{SEM}, and final uncertainties $(\sigma_{SEM}^2 + \sigma_{MC}^2)^{0.5}$, are indicated in green.

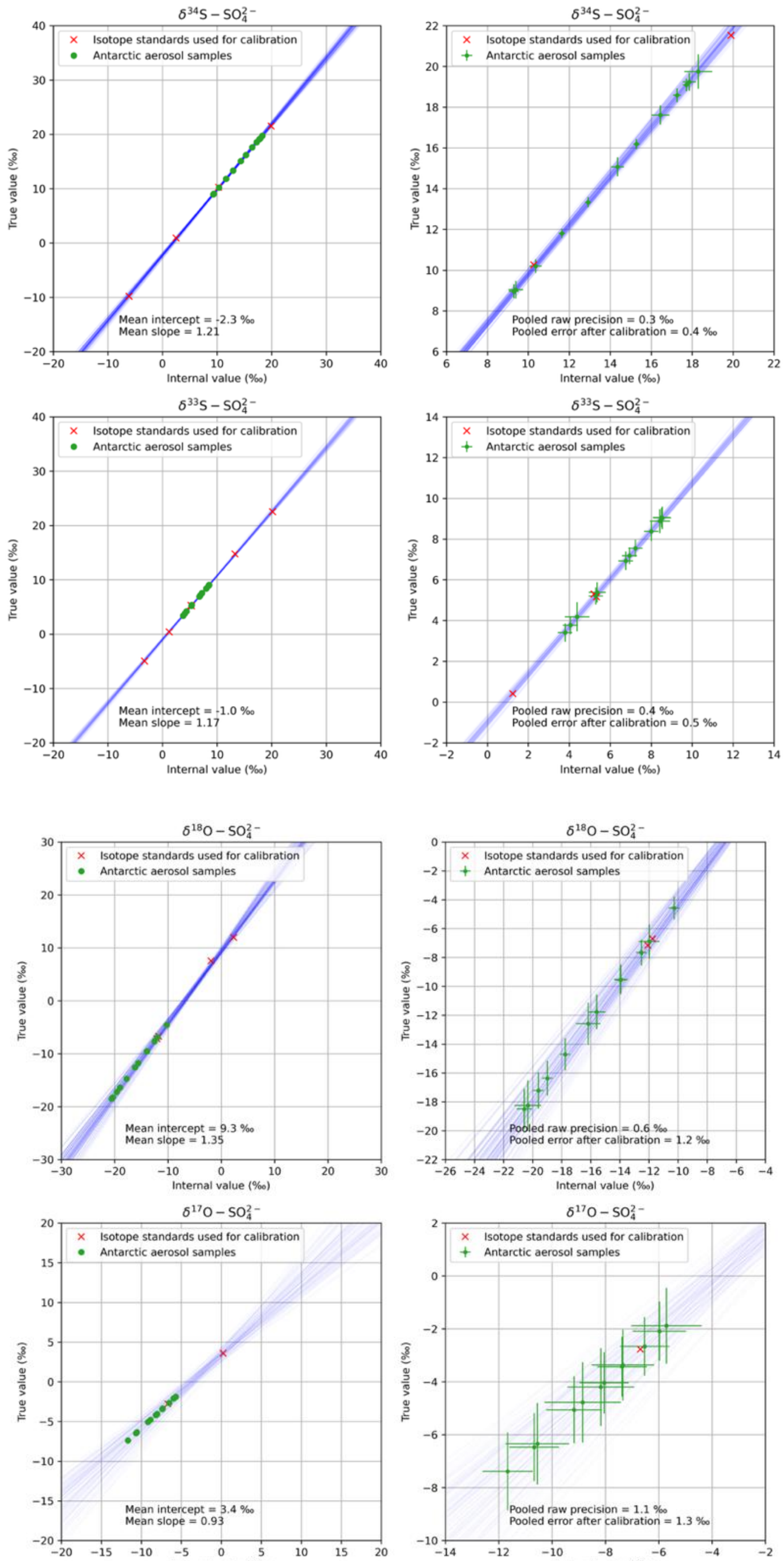

Figure S5: Monte-Carlo linear regressions used for calibration of HSO_4^- M_0 ESI-Orbitrap isotopologue δ-values. Right-hand plots are equivalent to left-hand plots except for magnified scales. Calibration standards are indicatd by red crosses, while values of Antarctic aerosol samples, their raw precisions, σ_{SEM}, and final uncertainties $(\sigma_{SEM}^2 + \sigma_{MC}^2)^{0.5}$, are indicated in green.

COMBINING ESI-ORBITRAP-MS NO_3^- M_0 AND *no-M_0* DATA

Text S4 Protocol for combining ESI-Orbitrap-MS NO_3^- M_0 and *no-M_0* data

The ESI-Orbitrap-MS NO_3^- M_0 experiment generates isotopologue δ-values relative to the unsubstitued M_0 isotopologue, while the ESI-Orbitrap-MS *no-M_0* experiment can access rarer isotopologues and generates isotopologue δ-values relative to a substitued isotopologue. The calibrated δ-values for the substituted isotopologue A obtained in the M_0 experiment, $\delta(A/M_0)_{M_0}$, is comparable to elemental isotope δ-value measurable by IRMS. However, a combination of calibrated M_0 and calibrated *no-M_0* data can be used to calculate an equivalent δ-value, using the relation

$$\delta(A/M_0)_{B_i} + 1 = [\delta(A/B_i) + 1][\delta(B_i/M_0) + 1], \qquad \text{(Equation S4)}$$

where B_i is a *bridging* isotopologue accessible in both M_0 and *no-M_0* experiments, $\delta(A/B_i)$ is measured in the *no-M_0* experiment and $\delta(B_i/M_0)$ is measured in the M_0 experiment.

For every substituted isotopologue A there are as many routes to calculate $\delta(A/M_0)_{B_i}$ using joint M_0 and *no-M_0* data as there are suitable *bridging* isotopologues B_i, with each route giving semi-independent $\delta(A/M_0)_{B_i}$ values and uncertainties. In order to fully exploit calibrated M_0 and calibrated *no-M_0* data for a series of natural samples, the joint M_0 and *no-M_0* $\delta(A/M_0)_{B_i}$ values, calculated via each suitable *bridging* isotopologue B_i, as well as the *M_0-only* $(A/M_0)_{M_0}$ measurement, where available, were calculated for every substituted isotopologue A, giving several $\delta(A/M_0)$ estimates for each sample. Mean values and a covariance matrix were generated using a Monte-Carlo scheme with 1000 iterations, drawing values from covarying normal distributions defined by the post-calibration δ-value uncertainties $\sigma_{tot}^2 = \sigma_{SEM}^2 + \sigma_{MC}^2$ and the non-negligible covariance between $\delta(^{18}O/^{15}N)$ and $\delta(^{17}O/^{15}N)$, derived from isotopologue abundances (see Notebook *Calculate-M0+noM0-Orbitrap-NO3.ipynb* at *DOI: 10.5281/zenodo.19495113*).

Averaged δ-values for each substituted isotopologue were calculated from the weighted average (weighting $1/\sigma^2$) of the set of estimates. Uncertainties were propogated analytically from the $\delta(A/M_0)$ value covariance matrix. It was observed in NO_3 acquisitions that

$\delta(^{17}O/M_0)_{M_0}$ was often systematically 1 – 2 ‰ lower than $\delta(^{17}O/M_0)_{^{15}N}$ and $\delta(^{17}O/M_0)_{^{18}O}$. ESI-Orbitrap-MS $\Delta^{17}O$-NO_3^- values agreed more strongly with IRMS measurements when only $\delta(^{17}O/M_0)_{^{15}N}$ and $\delta(^{17}O/M_0)_{^{18}O}$ were used in the weighted average calculation. As a result, the M_0 measurement of the NO_3^- ^{17}O peak, $(^{17}O/M_0)_{M_0}$, was not used in the calculation of any δ-values. Triple-isotope and clumped-isotope Δ-values were calculated from these weighted-average δ-values using the common formulae listed in Table 3 of the paper.

INTERFERENCE IN ESI-ORBITRAP-MS HSO_4^- *no-M_0* ANALYSIS

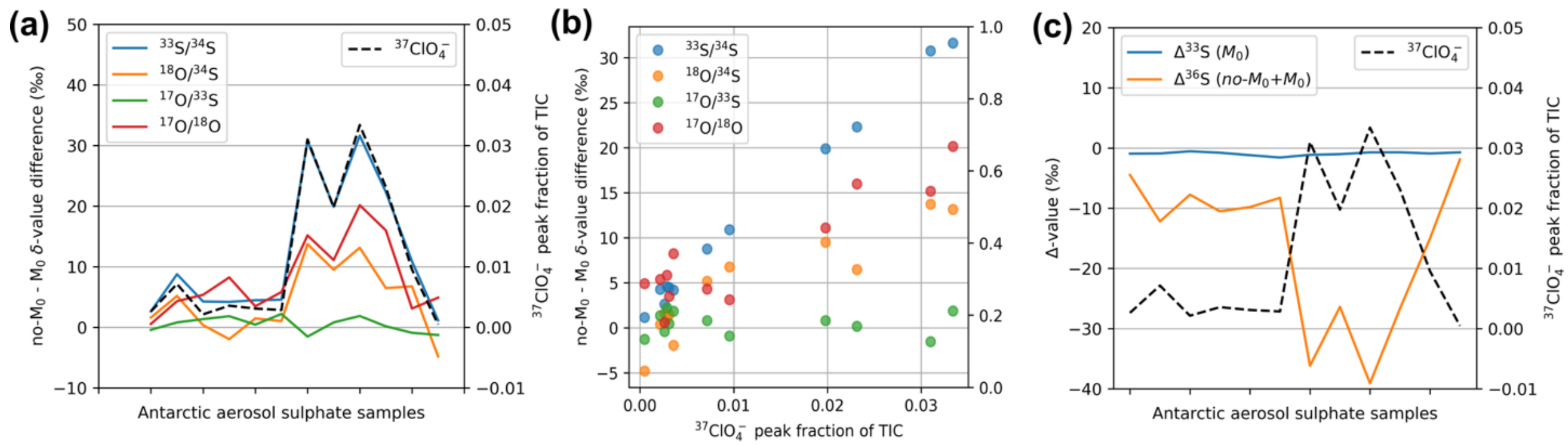

Figure S6: (a) Plots of the difference between HSO_4^- isotopologue δ-values measured in M_0 and *no-M_0* experiments, for four isotopologue ratios accessible in both M_0 and *no-M_0* acquisition modes, alongside the relative size of the contaminating $^{37}ClO_4^-$ peak, which was baseline-resolved from all HSO_4^- isotopologue peaks. (b) The corresponding cross-plots. M+2 isotopologue peaks (^{34}S and ^{18}O) appear smaller in the presence of ClO_4^- ions, while the M+1 isotopologues (^{17}O and ^{33}S) appear unaffected. (c) The M+4 ^{36}S isotopologue was also impacted: $\Delta^{36}S$, calculated via the ^{33}S basepeak showed a –40 ‰ excursion in contaminated samples, while $\Delta^{33}S$, measured in the M_0 experiment, remained close to zero.

ESI-ORBITRAP-MS STATISTICAL TESTING

Text S5 ESI-Orbitrap-MS statistical testing protocol

To generate the relative standard error (RSE), relative Allan deviation (RAD) and relative Hadamard deviation (RHD) plots in Figure 5 of the paper, 12 hour continuous ESI-Orbitrap-MS acquisitions were made. Here we detail the method used for acquisition and treatment of this data. We also present RSE, RAD and RHD plots for the isotopologue ratios not shown in Figure 5 of the paper.

Continuous data acquisition A digitally-controlled syringe pump (Thermo Scientific) and a 5 mL syringe (GasTight, Hamilton) were used to deliver a 4 μL/min flow of 45 μM acidified methanolic $NaNO_3$ or Na_2SO_4 solutions, via PEEK tubing (64 μm internal diameter), to the ESI-Orbitrap. The acquisition parameters listed in Table S1 were used for acquisitions up to 20 hours long.

Calculation of statistical metrics To ensure relevant statistical metrics, data acquired from continuous acquisitions were treated similarly to real sample data. The first hour of acquisition was discarded and spikes of extreme TIC (beyond 0.8 – 1.2 × local median TIC, 10 min rolling window) were discarded. RSE was calculated as $\sigma/\sqrt{n}$ for the first *n* scans, where σ is the isotopologue ratio standard deviation. RAD and RHD were calculated using the *oadev()* and *ohdev()* functions from the *AllanTools* Python package (https://github.com/aewallin/allantools/).

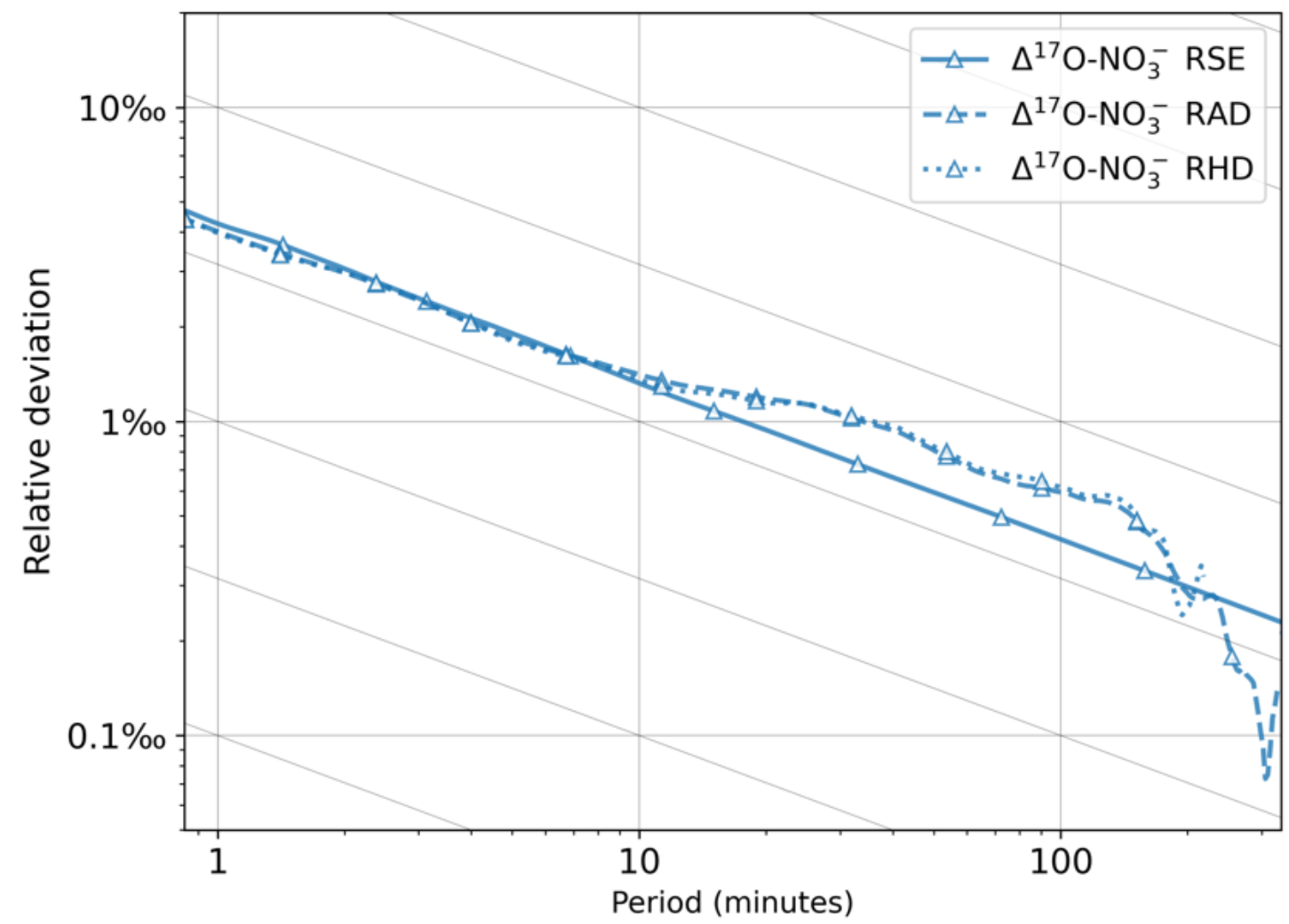

Figure S7: Relative standard error (RSE, solid lines), relative Allan deviation (RAD, dashed lines), and relative Hadamard deviation (RHD, dotted lines) for Δ^{17}O-NO_3 obtained in NO_3^- M_0 experiment.

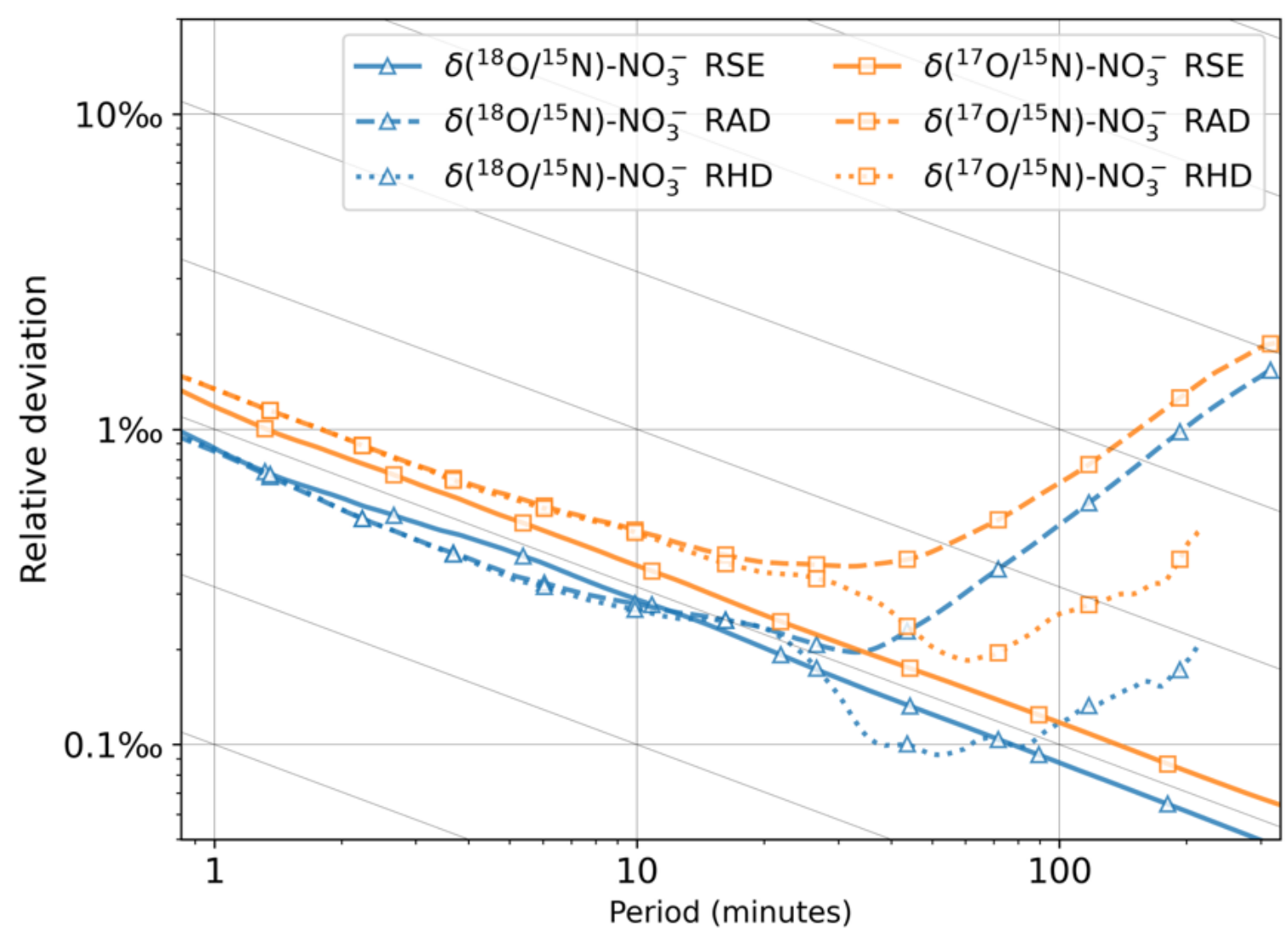

Figure S8: Relative standard error (RSE, solid lines), relative Allan deviation (RAD, dashed lines), and relative Hadamard deviation (RHD, dotted lines) for isotopologue ratios in NO_3^- *no-M_0* ESI-Orbitrap-MS experiments.

ESI-ORBITRAP-MS – IRMS – MC-ICP-MS METHOD COMPARISON

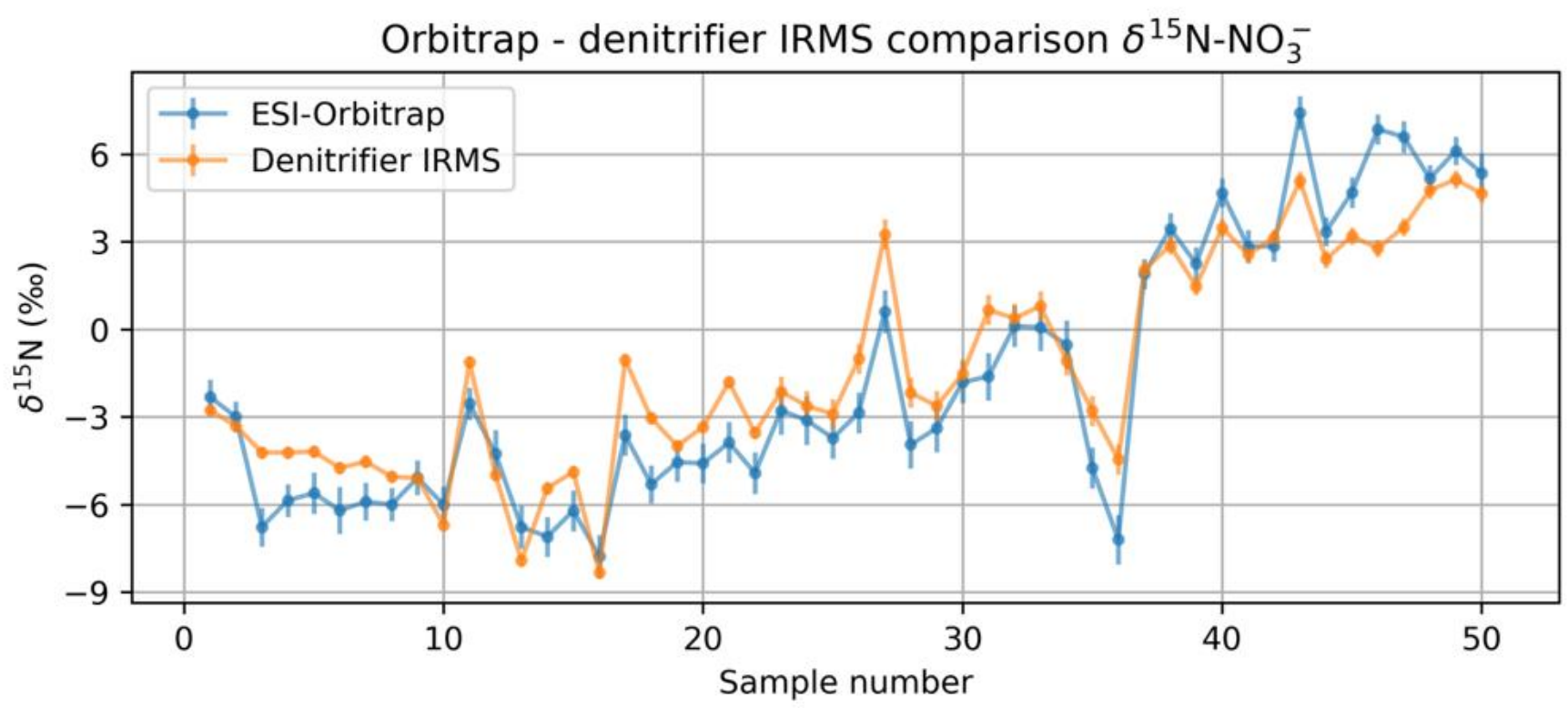

Figure S9: Line plot for δ^{15}N-NO_3^- ESI-Orbitrap-MS – denitrifier IRMS comparison

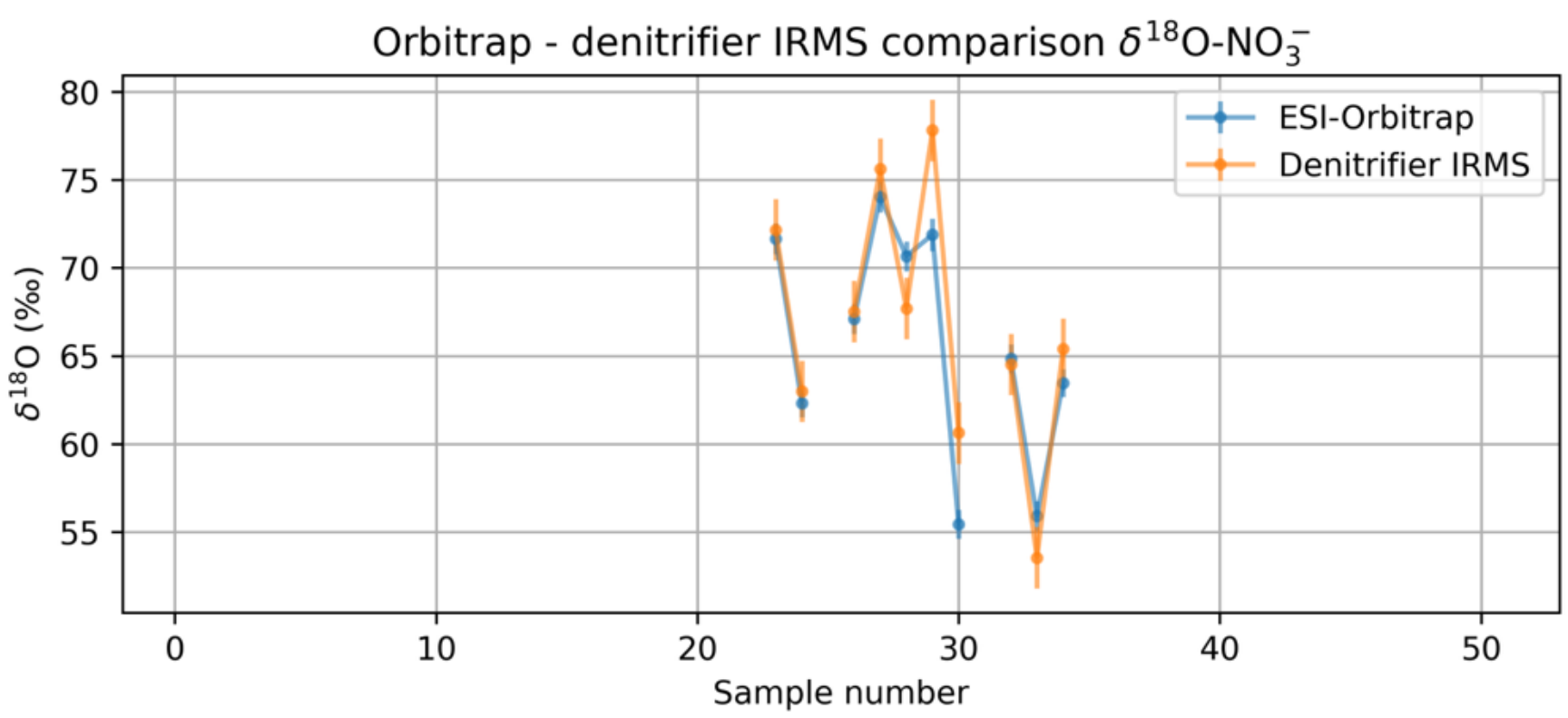

Figure S10: Line plot for δ^{18}O-NO_3^- ESI-Orbitrap-MS – denitrifier IRMS comparison

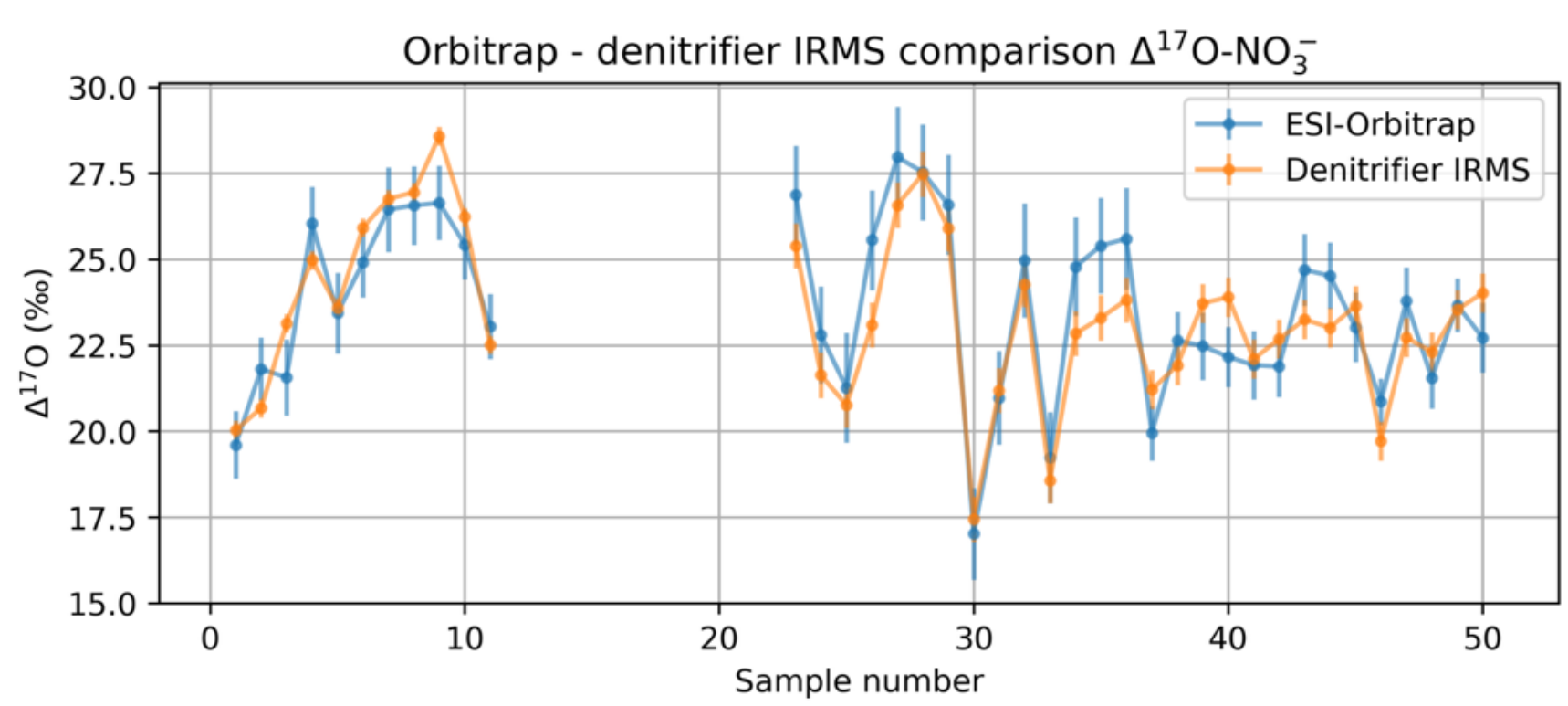

Figure S11: Line plot for $\Delta^{17}O\text{-}NO_3^-$ ESI-Orbitrap-MS – denitrifier IRMS comparison

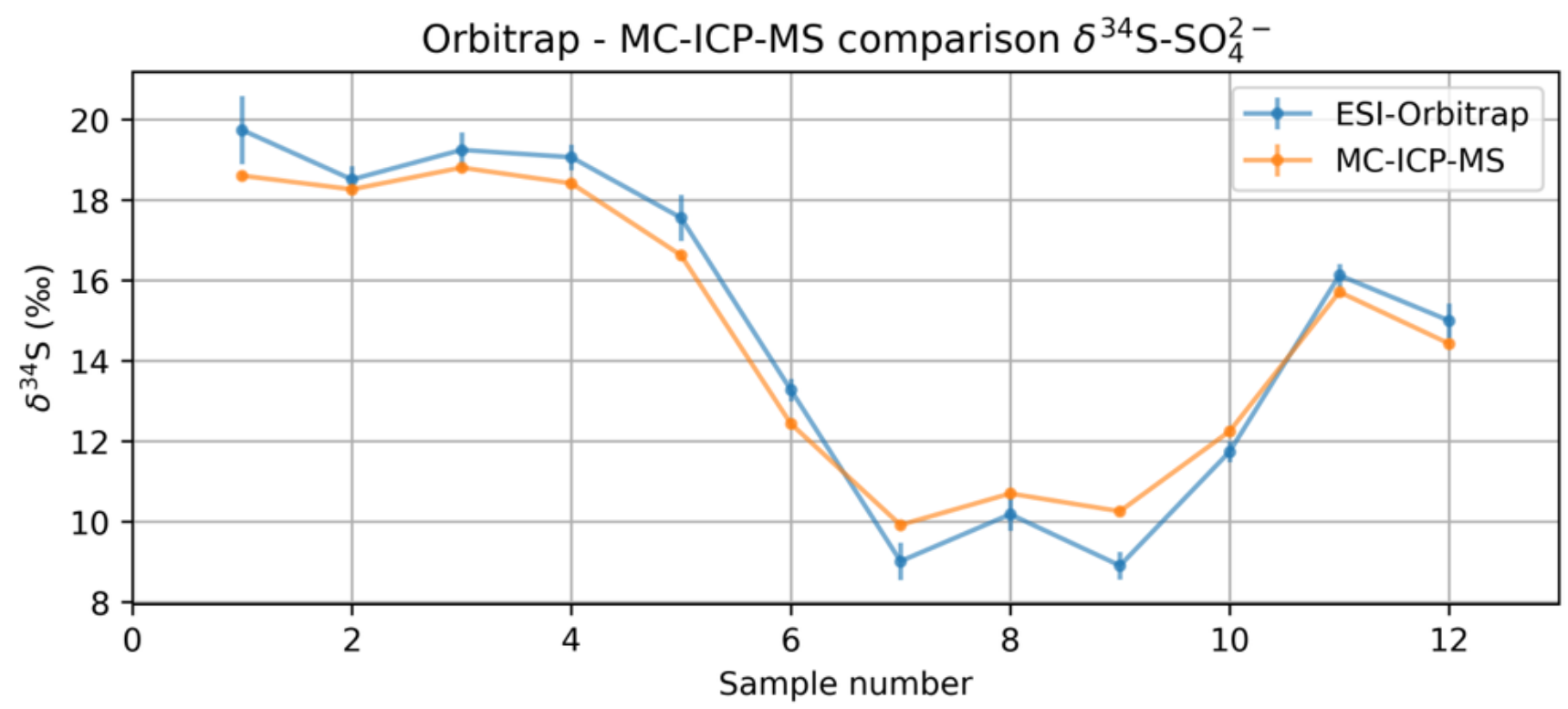

Figure S12: Line plot for $\delta^{34}S\text{-}SO_4^{2-}$ ESI-Orbitrap-MS – MC-ICP-MS comparison

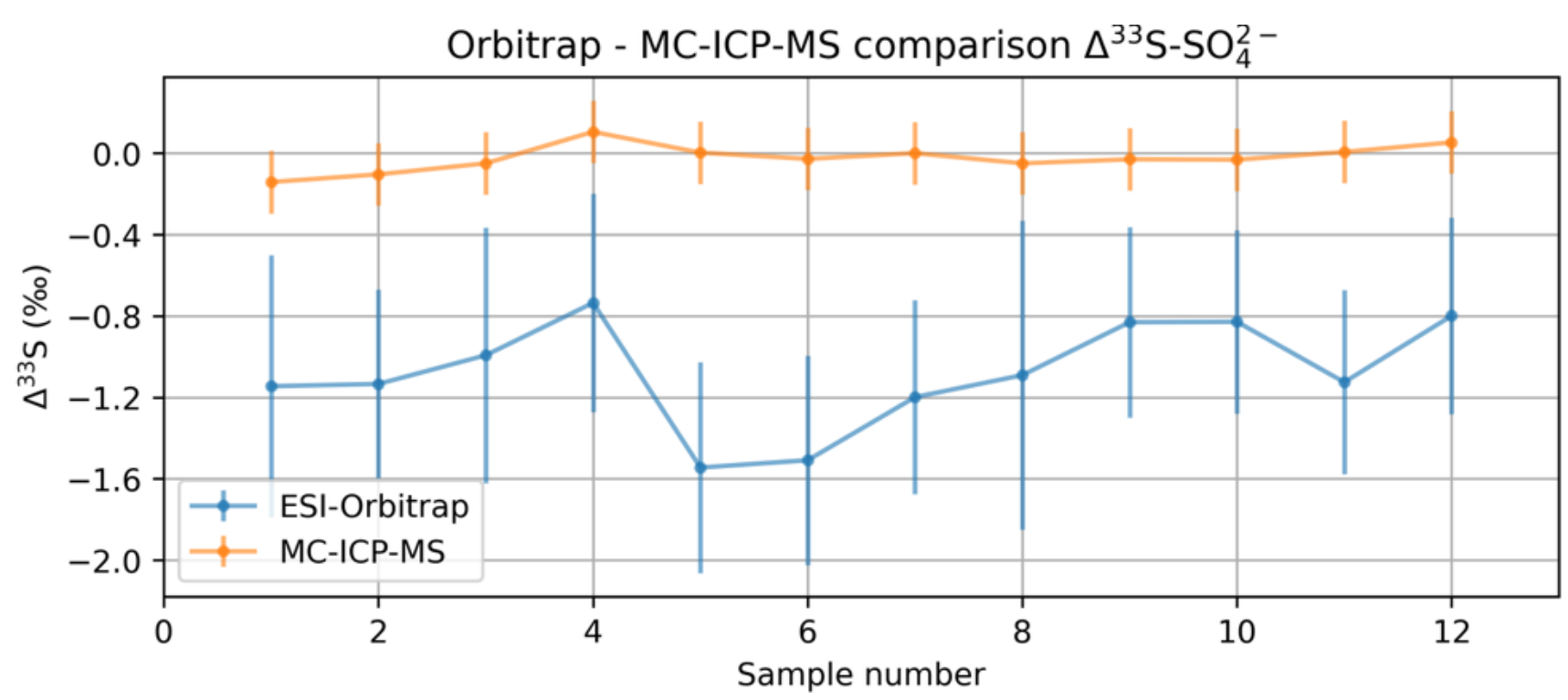

Figure S13: Line plot for $\Delta^{33}S\text{-}SO_4^{2-}$ ESI-Orbitrap-MS – MC-ICP-MS comparison

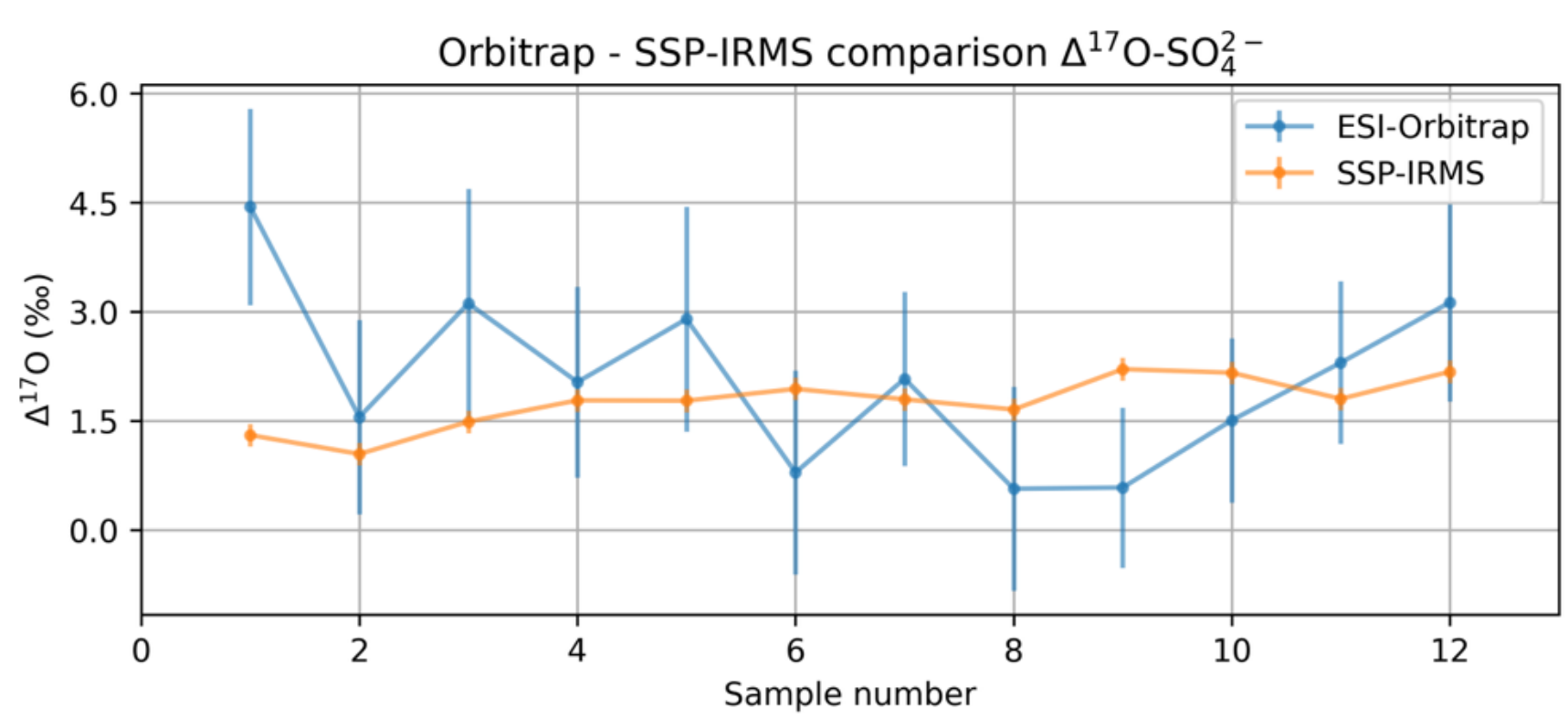

Figure S14: Line plot for $\Delta^{17}O\text{-}SO_4^{2-}$ ESI-Orbitrap-MS – SSP-IRMS comparison

Data set S1 ESI-Orbitrap-MS, denitrifier-IRMS, MC-ICP-MS and SSP-IRMS isotope measurements of Mont Blanc ice core samples and Antarctic aerosol samples used to generate the plots and statistics in Figure 7, Figures S9-S14, and Table 5 of the paper and are collated in a separate *.xlsx* file.

REFERENCES

Fry, B., Silva, S.R., Kendall, C., Anderson, R.K., 2002. Oxygen isotope corrections for online δ34S analysis. Rapid Commun. Mass Spectrom. 16, 854–858. https://doi.org/10.1002/rcm.651

Kornexl, B.E., Gehre, M., Höfling, R., Werner, R.A., 1999. On-line δ18O measurement of organic and inorganic substances. Rapid Commun. Mass Spectrom. 13, 1685–1693. https://doi.org/10.1002/(SICI)1097-0231(19990830)13:16<1685::AID-RCM699>3.0.CO;2-9

Schoenemann, S.W., Schauer, A.J., Steig, E.J., 2013. Measurement of SLAP2 and GISP δ^{17} O and proposed VSMOW-SLAP normalization for δ^{17} O and ^{17} O_{excess}. Rapid Commun. Mass Spectrom. 27, 582–590. https://doi.org/10.1002/rcm.6486

Stüeken, E.E., De Castro, M., Krotz, L., Brodie, C., Iammarino, M., Giazzi, G., 2020. Optimized switch-over between CHNS abundance and CNS isotope ratio analyses by elemental analyzer-isotope ratio mass spectrometry: Application to six geological reference materials. Rapid Commun. Mass Spectrom. 34, e8821. https://doi.org/10.1002/rcm.8821

Yu, Y., Sugden, P., Steele, R.C.J., Nuruzzama, M., Burke, A., 2025. Overcoming Polyatomic Interferences in Sulfur Isotope Measurement Using MC-ICP-MS. Geostand. Geoanalytical Res. https://doi.org/10.1111/ggr.70019
